# Interactions by 2D Gel Electrophoresis Overlap (iGEO): a novel high fidelity approach to identify constituents of protein complexes

**DOI:** 10.1186/1477-5956-11-21

**Published:** 2013-05-12

**Authors:** Masaaki Yoshigi, Stephen M Pronovost, Julie L Kadrmas

**Affiliations:** 1 Huntsman Cancer Institute, University of Utah, Salt Lake City, UT, 84112, USA; 2 Department of Pediatrics, University of Utah, Salt Lake, UT, 84112, USA; 3 Department of Oncological Sciences, University of Utah, Salt Lake, UT, 84112, USA

**Keywords:** PINCH, Interactome, Affinity purification, 2D-GE, DIGE, MudPIT

## Abstract

**Background:**

Here we describe a novel approach used to identify the constituents of protein complexes with high fidelity, using the integrin-associated scaffolding protein PINCH as a test case. PINCH is comprised of five LIM domains, zinc-finger protein interaction modules. In *Drosophila melanogaster*, PINCH has two known high-affinity binding partners—Integrin-linked kinase (ILK) that binds to LIM1 and Ras Suppressor 1 (RSU1) that binds to LIM5—but has been postulated to bind additional proteins as well.

**Results:**

To purify PINCH complexes, in parallel we fused different affinity tags (Protein A and Flag) to different locations within the PINCH sequence (N- and C-terminus). We expressed these tagged versions of PINCH both in cell culture (overexpressed in *Drosophila* S2 cell culture in the presence of endogenous PINCH) and *in vivo* (at native levels in *Drosophila* lacking endogenous PINCH). After affinity purification, we analyzed PINCH complexes by a novel 2D-gel electrophoresis analysis, iGEO (interactions by 2D Gel Electrophoresis Overlap), with mass spectrometric identification of individual spots of interest. iGEO allowed the identification of protein partners that associate with PINCH under two independent purification strategies, providing confidence in the significance of the interaction. Proteins identified by iGEO were validated against a highly inclusive list of candidate PINCH interacting proteins identified in previous analyses by MuDPIT mass spectrometry.

**Conclusions:**

The iGEO strategy confirmed a core complex comprised of PINCH, RSU1, ILK, and ILK binding partner Parvin. Our iGEO method also identified five novel protein partners that specifically interacted with PINCH in *Drosophila* S2 cell culture. Because of the improved reproducibility of 2D-GE methodology and the increasing affordability of the required labeling reagents, iGEO is a method that is accessible to most moderately well-equipped biological laboratories. The biochemical co-purifications inherent in iGEO allow for rapid and unambiguous identification of the constituents of protein complexes, without the need for extensive follow-up experiments.

## Background

Here, we present a multi-faceted approach to identify the constituents of a protein complex, using the extensively characterized integrin-associated scaffolding LIM protein PINCH [1] as a model molecule. The PINCH family of proteins has a modular domain structure consisting of 5 tandem LIM domains [2], cysteine and histidine-rich zinc fingers that are designed to act as stable protein interaction interfaces [2]. In mouse and humans there are two family members: PINCH1 and PINCH2 [3]. *Drosophila* has a single PINCH isoform, encoded by the *steamer duck* gene, with two direct and robust binding partners: Integrin-linked kinase (ILK) which binds to LIM1 of PINCH [4,5], and Ras Suppressor 1 (RSU1) that binds to LIM5 of PINCH [6,7]. Recent data on PINCH localization to integrin-rich adhesions in the absence of a targeting interaction with ILK has implied that PINCH may have additional partners to assist in its proper sub-cellular localization [8]. Thus, PINCH complexes are attractive as an archetype for purification, with two known partners to serve as positive controls, as well as the potential to identify novel interactors.

We previously analyzed PINCH-Protein A (PrA) complexes *via* MudPIT (Multi-dimensional Protein Identification Technology) mass spectrometry [6,9,10], a high sensitivity technique that generated an inclusive list of potential interacting proteins. Indeed, our analyses led to the identification of RSU1 [6], which after extensive biochemical characterization is now a well-accepted constituent of PINCH complexes [7,8,11,12]. Since our recent data suggests that PINCH may have (an) additional novel partner(s) to assist in its localization to integrin-rich sites [8], we revisited the list of candidate partners generated in the MudPIT analyses. As these candidates generally lack clear localization to integrin-rich sites, none were attractive for biochemical follow-up experiments.

As an alternative to pursuing candidates identified in the MudPIT analyses, we developed a novel two-dimensional gel electrophoresis (2D-GE) strategy to identify those proteins that consistently co-purify with PINCH under multiple conditions of tagging and expression. In this procedure, iGEO (interactions by 2D Gel Electrophoresis Overlap), fluorescent dyes typically employed for DIGE (Difference Gel Electrophoresis) are used to label differentially tagged PINCH complexes as well as control purified complexes. Upon simultaneous 2D-GE of these samples to separate the proteins based on both mass and isoelectric point, we readily identified in a single experiment gel spots that specifically co-purified with PINCH regardless of which affinity tag was employed. These spots of interest were subjected to in-gel tryptic digest and nano-LC-MS/MS [13] for identification.

To validate the iGEO approach, we compared the proteins identified to the candidates identified in previous MudPIT analyses. Under both the MudPIT and iGEO experimental strategies, we identified a core complex consisting of PINCH plus its confirmed direct interactors, recapitulating published biochemical data accumulated through years of extensive study [4-7,14-19]. Moreover, iGEO identified a number of proteins that specifically interacted with PINCH in *Drosophila* S2 cell culture. Cross-validated by the MudPIT data, we have demonstrated that our novel iGEO methodology can be effective in unambiguously determining the composition of multi-protein complexes.

## Results

### Mudpit data analyses

**Figure 1:**
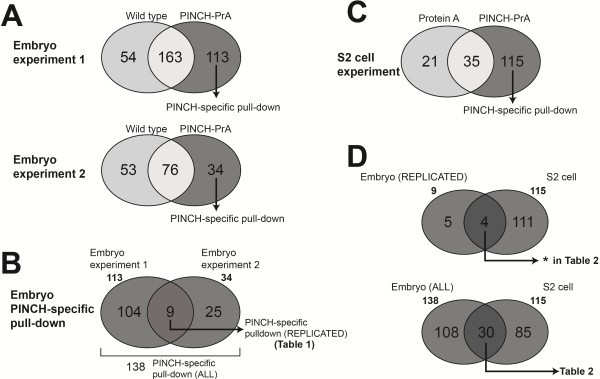
**Venn diagram analysis of MudPIT data sets. A**, Categorization of proteins identified in Embryo experiment 1 and 2. The complete PINCH-PrA data set includes a subset of proteins identified as ‘PINCH-specific pull-down’. The proteins identified in wild type embryos are pulled down non-specifically by purification matrix. A complete list of these proteins is available in Additional file 1: Table S1. **B**, The union of both Embryo experiment data sets constitutes an inclusive list of candidate PINCH binding partners. The intersection of the two data sets (dark shaded) represents candidates replicated in both experiments. **C**, Composition of proteins identified in *Drosophila S2* cell experiments. A complete list of these proteins is available in Additional file 2: Table S2. **D**, In the intersection of the S2 cell data set and the replicated proteins of the embryo data sets, 4 proteins were identified (indicated by * in Table 2). In the intersection of the S2 cell data set and the inclusive list of both embryo experiment data sets, 30 proteins were identified. The identities of these proteins are listed in Table 2.

We previously conducted a series of affinity purifications of PINCH-PrA complexes followed by MudPIT mass spectrometric analyses [6]. We identified RSU1 as a robust binding partner for PINCH in those experiments. Indeed, RSU1 was visible as a prominent band in silver stained 1D gels of PINCH-PrA complexes [6]. Here, we report and analyze the remainder of the MudPIT data sets in full. We performed two replicate pull-down experiments from extracts of wild-type (*w*^*1118*^) *Drosophila* embryos and PINCH mutant embryos rescued with a PINCH-PrA transgene. Using MudPIT analyses, we identified 113 and 34 proteins specific to the PINCH-PrA pull-down from the respective experiments (Figure 1A). A comprehensive list of all the proteins identified by MudPIT in PINCH-PrA and wild-type embryo data is available in Additional file 1: Table S1. As predicted, PINCH (Steamer duck) was prominently detected in both experiments. A comparison of PINCH-specific pull-down proteins between embryo experiment 1 and 2 revealed that nine proteins were replicated (Figure 1B) out of a total of 138 proteins detected in both experiments. In addition to established PINCH complex components ILK, parvin, and RSU-1, the nine proteins replicated between embryo experiment 1 and 2 included five novel candidate proteins (Table 1).

**Table 1:** Proteins identified in both Embryo 1 and Embryo 2 MudPIT data sets

			**Peptide coverage**	
**Flybase ID**	**Gene symbol**	**Name**	**Embryo 1**	**Embryo 2**
CG10302	*bsf*	bicoid stability factor	2.6%	2.9%
CG10504	*Ilk*	Integrin linked kinase (ILK)	36.6%	20.5%
CG14648	*lost*	lost	7.3%	16.6%
CG32528	*parvin*	parvin	55.6%	30.2%
CG4799	*Pen*	pendulin	12.8%	5.7%
CG5462	*scrib*	scribbled	2.8%	1.3%
CG7954	*stck*	steamer duck (PINCH)	48.7%	29.3%
CG8922	*RpS5a*	ribosomal protein S5a	15.8%	12.3%
CG9031	*ics*	icarus (RSU-1)	21.7%	17.6%

Complete MudPIT data sets of embryo experiments are available in Additional file 1: Table S1*.*

Because of the experimental design, the list of *Drosophila* embryonic proteins identified by the PINCH-PrA pull-down experiments may include candidates that bind to the PINCH-PrA fusion protein, but not PINCH alone. Therefore, a pull-down experiment was performed from extracts of S2 cells expressing either PINCH-PrA or Protein A alone to determine proteins that are co-purified in a PINCH-dependent manner. MudPIT analysis revealed 115 proteins specific to PINCH and independent of the Protein A affinity tag (Figure 1C). A comprehensive list of all the S2 cell proteins identified in the PINCH-PrA and Protein A control pull-downs is presented in Additional file 2: Table S2.

We compared the MudPIT data sets derived from both embryo and S2 cell experiments (Figure 1D). A complete list of all the proteins identified in these three experiments is presented in Additional file 3: Table S3. A stringent comparison using only candidates reproducibly identified in both embryo experiments (9 proteins) and the S2 cell candidates (115 proteins) yielded only 4 proteins that were reproducibly identified in embryos and in cell culture (Figure 1D). These four proteins included PINCH and its established interactome; ILK, Parvin and RSU1; and represent an unambiguous core complex. By comparing all the candidates identified in either of the replicate embryo experiments (138 proteins) to the candidates identified in S2 cells (115 proteins), we generated an inclusive list of 30 candidate proteins that are present in both the S2 cell data set and present in either of the embryo data sets (Figure 1D, Table 2). These are the strongest candidates, but they still require independent verification. As most of these candidates (other than Paxillin, which we presume to be recruited to PINCH complexes through its known interaction with Parvin and/or ILK [17,20]) lack a known localization to integrin-rich sites, we did not prioritize any of the MudPIT candidates for biochemical follow-up experiments.

**Table 2:** Proteins identified in two or more MudPIT analyses

			**Peptide coverage**			
**Flybase ID**	**Gene symbol**	**Name**	**Embryo E1**	**Embryo E2**	**S2 cell**	
CG10504	*Ilk*	Integrin linked kinase (ILK)	36.6%	20.5%	35.7%	*
CG10687	*Aats-asn*	Asparaginyl-tRNA synthetase	14.2%		6.5%	
CG10811	*eIF4G*	eukaryotic translation initiation factor 4G	2.8%		4.9%	
CG10938	*Prosα5*	Proteasome α5 subunit	18.0%		22.5%	
CG1100	*Rpn5*	Rpn5	9.0%		2.0%	
CG11271	*RpS12*	Ribosomal protein S12	51.1%		17.3%	
CG11522	*RpL6*	Ribosomal protein L6		6.6%	8.6%	
CG12030	*Gale*	UDP-galactose 4'-epimerase	8.9%		22.0%	
CG12233	*l(1)G0156*	lethal (1) G0156	19.5%		13.3%	
CG1404	*ran*	ran		10.6%	14.4%	
CG14207	*CG14207*		32.8%		24.0%	
CG18290	*Act87E*	Actin 87E	32.4%		26.3%	
CG18572	*r*	rudimentary	7.4%		3.7%	
CG2512	*αTub84D*	α-Tubulin at 84D	42.4%		30.2%	
CG31794	*Pax*	Paxillin	10.7%		11.5%	
CG3186	*eIF-5A*	eIF-5A	28.3%		14.5%	
CG32528	*parvin*	parvin	55.6%	30.2%	55.9%	*
CG32920	*Prx5*	Peroxiredoxin 5		18.4%	19.5%	
CG3416	*Mov34*	Mov34	15.7%		18.3%	
CG4046	*RpS16*	Ribosomal protein S16		12.8%	17.6%	
CG4183	*Hsp26*	Heat shock protein 26	38.9%		39.4%	
CG5119	*pAbp*	polyA-binding protein		6.5%	9.3%	
CG5289	*Pros26.4*	Proteasome 26S subunit subunit 4 ATPase		8.2%	11.2%	
CG6050	*EfTuM*	Elongation factor Tu mitochondrial	15.5%		8.6%	
CG7954	*stck*	steamer duck (PINCH)	48.7%	29.3%	66.6%	*
CG8882	*Trip1*	Trip1	16.3%		14.7%	
CG9031	*ics*	icarus (RSU-1)	21.7%	17.6%	63.2%	*
CG9412	*rin*	rasputin		1.2%	4.4%	
CG9674	*CG9674*		1.8%		1.9%	

Proteins with an asterisk (*) in the right column were identified in all three data sets, indicating they are *bona fide* constituents this protein complex.

### 2D-GE data collection and analyses

In an attempt to identify additional components of the PINCH interactome, we developed a novel 2D-GE strategy. Using PINCH-PrA and Flag-PINCH, we purified PINCH-associated protein complexes from S2 cell lysates. We devised an application called iGEO that employs tri-color fluorescent sample labeling to simultaneously identify 2D-GE protein spots specifically co-purifying with two differently tagged versions of PINCH. The foundation of the method is an alternative utilization of CyDye fluorophores (GE Healthcare), commonly used for 2D-DIGE analyses [21].

**Figure 2:**
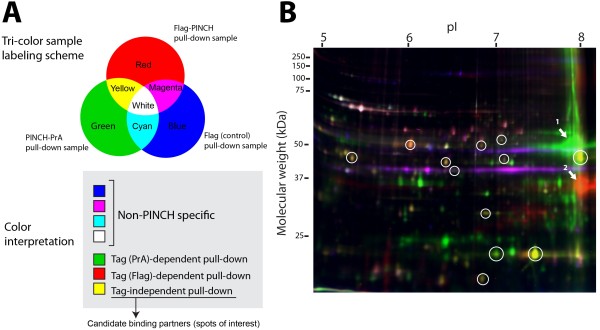
**iGEO multi-color labeling scheme to identify PINCH-specific protein interactions. A,** Tri-color iGEO labeling scheme to identify tag-independent PINCH binding partners in a single 2D gel. We applied CyDyes used for 2D-DIGE for protein labeling of Flag pull-down (red, Cy5), Protein A pull-down (green, Cy3), and Flag control (blue, Cy2). Spots containing both red and green hues, but devoid of blue hue are the spots of interest. **B,** A representative image of a 2D gel with the tri-color sample labeling. Twelve spots (circled in white) showed both red and green hues, but lacked blue hue. Arrows indicate bait proteins, PINCH-PrA (arrow 1), and Flag-PINCH (arrow 2). A source file of the gel image that allows inspection of signal intensity on each color channel is available as Additional file 4: Figure S1.

In a typical labeling protocol, PINCH-PrA complexes, Flag-PINCH complexes, and vector (Flag or Protein A) associated samples were labeled by green Cy3 dye, red Cy5 dye, and blue Cy2 dye, respectively (Figure 2A). After quenching excessive dyes, all the dye-labeled samples were combined and run on a single 2D gel. We quantified signal intensity of individual spots using plugins available for ImageJ, and manually created a spreadsheet file of spot coordinates as well as signal intensity of each color channel. Using an RGB color scheme (Figure 2A), fluorescent 2D gel spots containing blue signals (*i.e.* blue, magenta, cyan, and white spots) are proteins present in the vector control samples that co-purify independently of PINCH. Spots containing only green or red hue are proteins present only in either PINCH-PrA-associated or Flag-PINCH-associated samples, respectively, indicating they may associate with PINCH in a tag-dependent manner of little biological significance. Flag-PINCH samples repeatedly showed 100–120 associated spots (red channel) while PINCH-PrA samples showed 40–50 spots (green channel). Flag control samples showed approximately 40–50 spots (blue channel) while Protein A control consistently showed 10–15 spots (data not shown). This demonstrates that the affinity tag-interactome is partially dependent upon the type of the tag employed, and the Flag tag produces abundant tag-dependent interactions. In the iGEO analysis, yellow spots, red spots with minor green hue, or green spots with minor red hue are derived from both Flag-PINCH and PINCH-PrA pull-down samples, indicating these proteins associate with PINCH in a tag-independent manner. These spots represent strong PINCH interactome candidates, and are therefore spots of interest.

In a representative tri-color fluorescence gel, we identified a total of 12 tag-independent spots of interest, containing both green and red hue, exclusive of blue hue (Figure 2B, white circles). Raw images that allow detailed inspection of signal intensity on each color channel are available as Additional file 4: Figure S1. By design, PINCH-PrA and Flag-PINCH baits appeared as robust green and red spots, respectively (Figure 2B, indicated by arrows 1 and 2). As predicted by the appended tag, these spots exhibited an increase in molecular weight and shift in isoelectric focus point relative to native PINCH. The locations of Flag-PINCH and PINCH-PrA spots were confirmed using anti-PINCH antibody and 2D immunoblotting analysis (data not shown). The coordinates of the spots of interest were cataloged using approximate isoelectric point and molecular weight in order to verify reproducibility in subsequent individual gel experiments. As recommended by DIGE protocols, we repeated the tri-color fluorescence 2D-GE experiments with dye swap labeling, which eliminated the possibility of dye color-dependent interactions (data not shown).

**Figure 3:**
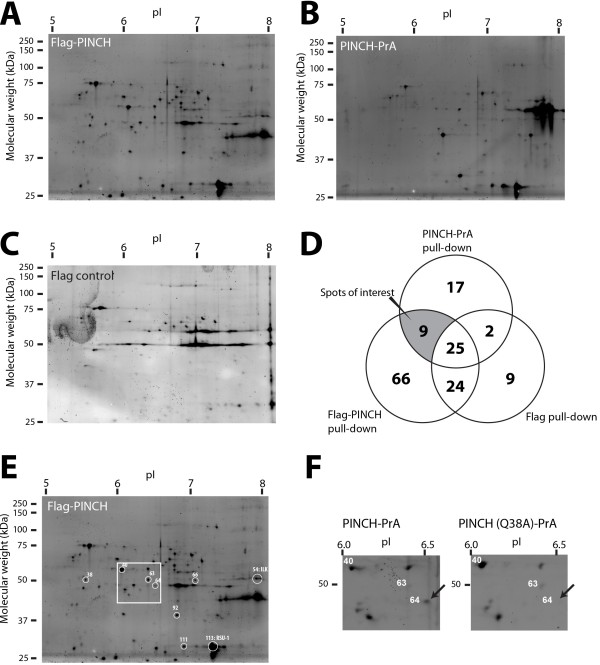
**Individual gel analysis of affinity tagged PINCH protein complexes.** Separate pull-down samples were analyzed in individual 2D gels, and visualized using Deep Purple fluorescent stain: Flag-PINCH (**A**), PINCH-PrA (**B**), and Flag control (**C**). Pull-down experiments were repeated in triplicate, and compared in a Venn diagram (**D**). Nine spots appeared reproducibly regardless of the tag employed, and were absent in control gels. **E,** A representative Flag-PINCH gel showing nine PINCH-specific spots of interest (circled in white). The numbers are derived from the master catalog used to match coordinates of all gels. Spot 54 and 113 were identified as ILK and RSU-1 by 2D immunoblot, respectively (see Additional file 5: Figure S2). The boxed area is highlighted in panel F. **F,** Independent pull-down experiments were performed comparing PINCH to PINCH^Q38A^, which disrupts the PINCH-ILK interaction. MS analysis revealed that spot 64 contained Parvin, and spot 64 is absent in the PINCH^Q38A^ purification, as indicated by the arrow.

We validated the iGEO analysis by running confirmatory 2D-GE pull-down experiments in triplicate, analyzed by individual gels stained with fluorescent Deep Purple dye to assess the reproducibility of the spot patterns. Representative patterns of 2D gels of individual Flag-PINCH, PINCH-PrA, and control samples purified from S2 cells are shown in Figure 3. We cataloged all the spots of individual gels, based on the location of spots present on Flag-PINCH gels (with the highest number of spots observed) as a guide. Then, the coordinates of all spots were compared to record their occurrence in individual gels. Venn diagrams were used to compare the gel spots that appeared consistently in triplicate (Figure 3D), resulting in 9 gel spots absent in control samples that reproducibly appeared in both the iGEO analysis as well as the individual Flag-PINCH and PINCH-PrA associated samples (Figure 3D,E).

We attempted to match these spots to either known PINCH interactors or to the protein candidates identified by MudPIT. Of the 9 spots identified, we verified the spot location of established PINCH-binding partners, ILK and RSU1 using 2D immunoblotting (Additional file 5: Figure S2, Additional file 6: Method S1). The remaining seven spots of interest were subjected to nano-LC-MS/MS analysis. Table 3 shows the identity of the proteins contained in the seven spots (raw data of MASCOT search results are available in Additional file 7: MASCOT raw data). Spot number 64 was identified as Parvin, an established ILK binding partner, also reproducibly identified by MudPIT. Notably, this spot was completely absent when PINCH^Q38A^-PrA was used as bait in a separate experiment (Figure 3F). PINCH^Q38A^ disrupts the PINCH-ILK interaction [8] and thereby also eliminates the association of Parvin, a direct ILK binding partner [22]. Spot number 111 was identified as an isoform of RSU1 with a shifted isoelectric point. The remaining five proteins were either proteasome components or chaperones: Droj2, ERp60, Pros35, Rpt1, and Rpt3 (Table 3). We cross-checked these candidates with the MudPIT data sets. These five proteins were not present in the reproducible candidates of interest from the MudPIT data sets (Table 1 and Table 2). However, four of the five proteins (spot number 38, 40, 66, and 92) were specifically identified in the MudPIT analyses, but only in the S2 cell data set (see proteins highlighted in yellow in Additional file 2: Table S2). One protein (spot number 63, Rpt1) was not specifically identified in any of the MudPIT data sets, but nonetheless co-purified with PINCH under the two independent sets of conditions used in the iGEO analysis. To explore the possibility that these proteins interact with PINCH only in cell culture, we repeated the 2D-GE analyses using purified PINCH-PrA and PINCH-Flag complexes isolated from adult fly lysates. We found that ILK, RSU1 (both isoforms) and Parvin reproducibly co-purified with both tagged versions of PINCH in adult lysates, but the other five spots were absent from the 2D gels (data not shown). Thus, these chaperone and proteasome proteins likely reflect cell culture-specific binding partners that may associate with tagged and overexpressed PINCH to assist in its folding and turnover. In summary, using iGEO, we confirmed all previously identified PINCH interacting proteins, and uncovered five additional proteins that specifically co-purify with PINCH in S2 cell culture.

**Table 3:** Proteins of interest identified by iGEO and nano-LC-MS/MS analysis, cross-referenced to their presence in the MudPIT data sets

						**Presence in MudPIT data set**		
**Spot ID**	**Flybase ID**	**Protein ID**	**Function**	**MW**	**pI**	**Embryo 1**	**Embryo 2**	**S2 cell**
38	CG16916	Rpt3	26S Proteasome Subunit	47	5.0	-	-	+
40	CG8983	ERp60	Protein disulfide isomerase homolog	55	5.6	-	-	+
63	CG1341	Rpt1	26S Proteasome Subunit	48	5.8	-	-	-
64	CG32528	Parvin	Parvin	42	6.6	+	+	+
66	CG8863	Droj2	DnaJ-like-2 chaperone	45	6.4	-	-	+
92	CG4904	Pros35	Proteasome 35kDa subunit	31	6.5	-	-	+
111	CG9031	RSU-1	RSU-1 isoform	31	6.6	+	+	+

Raw data of MASCOT search results are available in Additional file 7: MASCOT raw data.

## Discussion

There are many techniques available to identify protein-protein interactions, and all of them have both strengths and shortcomings [23]. Affinity tags, often coupled with mass spectrometric analyses, have been a prevalent way to purify protein complexes in the last decade because of the ease of purification and convenience [24-27]. Drawbacks of tagging technologies include; 1) the presence of a tag may have an impact on protein expression, turnover, or localization, 2) a tagged protein may have different partner binding characteristics as compared to the native form. Hence, the altered characteristics of the tagged protein may lead to false discovery of spurious interactions, or to the exclusion of *bona fide* interactions [28,29]. Given these considerations, when we set out to identify the interactome of the LIM domain scaffolding protein PINCH in *Drosophila*, we took a multi-faceted approach. We used different affinity tags fused to PINCH in different locations, expressed both in cell culture and in intact *Drosophila*. In order to have confidence in a PINCH interaction, we required that the protein co-purify with PINCH via more than one approach. After purification, we analyzed the PINCH-containing complexes by iGEO, our novel 2D-GE overlap methodology to identify proteins that specifically co-purified with PINCH under two independent sets of tagging conditions. We validated the robustness of the proteins identified by iGEO both by comparison to existing MudPIT mass spectrometric data and by confirmatory individual 2D-GE. Each tagging, expression, purification and analysis strategy generated a sizable number of candidate interacting proteins. However, only previously validated partners for PINCH (ILK, RSU1 and Parvin) were identified in every experiment, regardless of the strategy employed, indicating that these four proteins form an unambiguous core protein complex. Additionally, iGEO identified five proteasome and chaperone proteins that specifically associate with PINCH in S2 cell culture, regardless of the affinity tag employed or its location. Drawing from our analyses, the following are specific points of discussion for each component of our strategy.

### Tag selection and placement

When appended to PINCH, affinity tags give a rich cohort of co-purified proteins, as evidenced by 2D gel patterns (Figure 2B, Figure 3) and the MudPIT data sets. However, the bulk of these proteins represent either artifacts of the sample preparation or tag-specific binding partners. The control data from our MudPIT analyses allowed us to generate a list of common contaminants that interact with the agarose beads (shown in Additional file 1: Table S1), as well as proteins that interact with the Protein A tag (shown in Additional file 2: Table S2). We regard the lists of non-specific interactors in this study, in addition to those from previous studies [30,31], as a useful cross-reference when similar efforts to purify interaction partners are undertaken.

Because protein-protein interactions can be disrupted by the presence of an affinity tag, *bona fide* interactions could be overlooked when affinity tags are employed. Using an alternate fusion site for a second tag may allow for the identification of these partnerships. The strategy of using multiple tags to increase specificity during purification has been described in methods like Tandem Affinity Purification [32], and interactomes by Parallel Affinity Capture [31]. A novel aspect of our experimental design was in independently fusing affinity tags to two different locations within the PINCH sequence. However, our stringent iGEO conditions for identifying PINCH complex components include only proteins that interact with both PINCH-PrA and Flag-PINCH. Of note, it is important to retain all of the candidates identified, since any *bona fide* complex components whose association with PINCH was disrupted in one of the purification schemes by the choice of tag or its location would be overlooked in these analyses. To identify interactions that are undetected for this reason, a more extensive series of parallel purifications in which the tags are systematically varied could be fruitful.

### Biological starting material

Selecting an appropriate biological source is key for purifying biologically significant protein complexes. It can sometimes be difficult to choose a cell or tissue that expresses all of the relevant candidates, particularly if they are spatially or temporally limited in their expression. Moreover, if the complexes of interest form weak or transient interactions, are low abundance, or are poorly soluble, the limits of biochemistry may preclude the ability to detect the interactions, even with optimal source material. *Drosophila* S2 cultured cells are a convenient and readily accessible material for biochemical purification. In the majority of our iGEO experiments, we expressed tagged PINCH in S2 cells, which co-express endogenous PINCH. We also routinely purify PINCH complexes from *Drosophila* embryo or adult lysates because of the high complexity of proteins expressed at these developmental stages. In the fly, we expressed the tagged PINCH transgenes at native levels to fully rescue a genetic background in which all endogenous PINCH is eliminated. This ensures that complex formation is influenced neither by overexpression of PINCH, nor by tagged PINCH competing for partners with endogenous PINCH. PINCH partners identified from a variety of biological source materials indicate a binding interaction with PINCH in multiple cellular contexts. However, it is important to retain all identified candidates as possible proteins for further study, as *bona fide* partners may not necessarily be expressed in each of the samples analyzed.

Our iGEO analyses in S2 cells led to the identification of five novel protein partners that specifically associated with both PINCH-PrA and Flag-PINCH, four of which were also identified in the PINCH-PrA S2 cell MudPIT data. This serves to validate the utility of the iGEO approach. These five proteins are all either chaperones or proteasome components that were not found to associate with PINCH in *Drosophila* embryo lysates analyzed by MudPIT or in adult fly lysates analyzed by 2D-GE. This strongly suggests that overexpression of tagged PINCH in cell culture may promote protein interactions that assist in the expression, folding, or turnover of a non-native target protein expressed at non-physiological levels. To test this notion, different cell culture conditions might be tested (*e.g.* 3D culturing) such that PINCH interacting partners are altered. However, the novel interactions uncovered this way may have a limited biological significance.

### Analysis of purified complexes

We previously analyzed PINCH-PrA complexes purified from both *Drosophila* embryo lysates (2 replicates) and S2 cell lysates by MudPIT, a shotgun mass spectrometric method. PINCH-RSU1 binding was initially identified through these MudPIT analyses [6], and this protein interaction is now widely recognized and studied by other researchers [7,8,11,12]. MudPIT provides extremely high sensitivity for identifying protein constituents from a complex protein mixture, and is limited only in identifying proteins that do not give an adequate number of suitable cleavage fragments for mass spectrometric identification. In the embryo MudPIT analyses presented here, reproducibility was not as high as we might have predicted for the replicate embryo samples—only 9/138 proteins from the combined PINCH-specific candidates were identified in both samples. This underscores the inherent variability in the proteins purified, even when the preparation of replicate samples is closely matched, and reinforces the need for replicate experiments. Another consideration is that MudPIT generates a sizeable list of candidates that often require extensive biochemical follow up experiments to validate their inclusion within a protein complex. Shotgun proteomics methodologies continue to be improved and developed to increase the level of confidence in candidates for follow-up experiments. For instance, Stable isotope labeling by amino acids in cell culture (SILAC) mass spectrometric analysis detects differences in protein abundance among differentially labeled samples using non-radioactive isotopic labeling. It is a popular method for generating quantitative data on the composition of protein complexes [30,33]. In fact, the direct PINCH binding partner ILK has been subjected to SILAC-mass spectrometric analysis [34], uncovering new roles for ILK in mitotic spindle organization [35] and microtubule-dependent caveolar trafficking [36] deduced from the novel protein partnerships identified. The expense of isotope labeling in cell culture, the technical challenges of labeling in intact animals, along with the specialized mass spectrometric analyses required for the labeled proteins are some of the challenges to widespread use of SILAC technology.

As a complementary approach, we turned to 2D-GE to identify additional components of the PINCH interactome. We developed a novel application of DIGE technology called iGEO. In DIGE, the proteomes from two different conditions are labeled and then compared to look for a small number of differences amidst a vast majority of conserved proteins spots. In contrast, iGEO uses CyDye labeling to identify the overlap in the spots that co-purify with PINCH from two independent purification strategies, amidst a background of spots that purify in a non-specific manner. With current protocols that increase sensitivity via fluorescent stains and laser scanning, 2D-GE produces reliable and reproducible spot patterns from pull-down samples. 2D-GE analysis is limited in resolving extremely acidic, basic, or high molecular weight proteins, and reproducible 2D analysis is still dependent upon the technical expertise of the operator [37]. Even in light of the limitations of 2D-GE, iGEO identified all known PINCH complex components plus five novel proteins that specifically associated with PINCH in S2 cells, validating the iGEO approach. The iGEO approach is well within the capabilities of most biological laboratories. DIGE dyes are becoming more affordable and 2D-GE equipment is broadly available, as is access to a laser scanner and services for mass spectrometric identification of proteins from 2D gel slices. iGEO lacks the sensitivity of shotgun strategies but yields a small number of protein spots whose inclusion in the complex has already been robustly verified by two independent biochemical purifications.

## Conclusions

Given the modular LIM domain structure of the PINCH protein and recently published data suggesting new PINCH partners should participate in its subcellular localization [8], additional partners for PINCH are likely to exist. If we are to identify them, we will have to circumvent the limitations of the purification strategies mentioned above. As we explore additional purification strategies, including but not limited to affinity tag choice, source materials, or solubilization conditions, we may confirm more *bona fide* PINCH partners in future studies. Moving forward, iGEO will be a key analysis tool in our efforts. Moreover, a gel electrophoresis-based proteomics strategy like iGEO is a robust, high-fidelity, highly accessible approach for laboratories working to identify protein-protein interactions.

## Methods

### Expression constructs

Affinity tags were fused to either the N-terminus of the PINCH sequence (labeled as tag-PINCH) or to the C-terminus (labeled as PINCH-tag). Plasmids for S2 cell expression were constructed in pMT/V5/HisA (Invitrogen), containing a metallothionein promoter for CuSO_4_ induction. pMT-PINCH-PrA has been previously described [6] and the PINCH coding sequence was excised to generate the pMT-PrA control plasmid. A version of PINCH that cannot bind properly to ILK [8], pMT-PIN^Q38A^-PrA, was made *via* site directed mutagenesis of pMT-PINCH-PrA. pMT-Flag-PINCH was generated by adding a 3xFlag cassette (from p3xFlag-CMV-10, Sigma-Aldrich) to the N-terminus of the PINCH coding sequence in pMT-PINCH [6]. The pMT-Flag control plasmid was made by inserting the same 3xFlag cassette into pMT/V5/HisA. Upon induction in *Drosophila* S2 cells, the expression level of tagged PINCH is greater than endogenous PINCH expression.

The pCasPIN-PrA plasmid for transgenesis of *Drosophila* has been previously described [6]. It contains a genomic fragment encompassing the PINCH coding region plus 3 kB of upstream PINCH promoter, with a C-terminal fusion to the Protein A coding sequence, in the plasmid pCaSpeR. pCasPIN-Flag was generated by excising the Protein A sequence from pCasPIN-PrA and replacing it with a 3xFlag cassette [8]. Transgenic *Drosophila* resulting from genomic insertion of these plasmids were made in a wild type *w*^*1118*^ genetic background using standard techniques, then crossed into a PINCH null genetic background (*stck*^*17*^*/stck*^*18*^) [5,38]. Expression of the PINCH-Flag transgene is comparable to endogenous levels of PINCH [8] and fully rescues the embryonic lethality associated with PINCH loss-of-function. Wild type *w*^*1118*^ flies bearing no transgene were used as the negative control in embryo experiments.

### Pull-down sample preparation

*Drosophila* S2 cells (1.5 × 10^9^ cells/sample) or 0–24 hour *Drosophila* embryos (10 g/sample) were Dounce homogenized in 10 ml TLB (Triton Lysis Buffer: 0.1% Triton, 50 mM Tris–HCl pH 7.9, 150 mM NaCl) plus standard protease inhibitors. Lysates were centrifuged 10 minutes at 16,000 × g and resulting supernatants were collected and filtered (0.45 μm) before use. IgG Sepharose 6 Fast Flow (Amersham) or Anti-Flag M2 agarose (Sigma) beads (100 μl/sample) were equilibrated with three 1 ml washes each of 100 mM glycine pH 3.5, then TLB. Supernatants and beads were incubated for 1.5 hours at 4°C on a rocking platform. Beads were washed in 3× 10 ml TLB (batch wash) followed by 3× 1 ml TLB in a Bio-Rad BioSpin chromatography column. Complexes were eluted with 4× 300 μl 100 mM glycine pH 3.5. Elutes were immediately neutralized with 1:10 volume 1M Tris–HCl pH 8.8. Using a small portion of the purified material, effective pull-down was confirmed by one dimensional SDS-PAGE with silver stain.

For MudPIT, purified PINCH-PrA associated protein preparations were TCA precipitated by standard methods. For 2D-GE, pull-down samples were processed by TCA-acetone precipitation/recovery (Bio-Rad, 2D Clean-up Kit) to eliminate conductive materials and make the samples compatible with 2D-GE protocols. After TCA-acetone precipitation, samples were resuspended in 7M urea, 2M thiourea, 4% CHAPS buffer.

### MudPIT mass spectrometric analyses

MudPIT analysis was performed in collaboration with John R. Yates’ laboratory (The Scripps Research Institute), and detailed experimental procedures have been previously described [6,39]. Briefly, TCA precipitated PINCH-PrA complexes were resuspended in Tris buffer, 8M urea, pH 8.6, reduced, and alkylated. Complexes were endoproteinase Lys-C digested (4 h), diluted to 2M urea, and digested with trypsin overnight [9]. Peptide mixtures were loaded onto a triphasic LC/LC column and analyzed as described [39]. Tandem mass spectra were analyzed using SEQUEST and the *Drosophila* sequence database with threshold values of 1.8 (+1), 2.8 (+2), and 3.5 (+3) [9].

### iGEO and two dimensional gel electrophoresis (2D-GE)

We used a two-stage approach to analyze spot patterns of 2D gels. In the initial screening stage, we devised a novel multi-color CyDye labeling strategy called iGEO (CyDye DIGE Fluor, Minimal labeling dyes, GE Healthcare) to run three samples concurrently in a single 2D gel to minimize gel-to-gel variation in spot locations. PINCH-PrA, Flag-PINCH, and control pull-down samples from S2 cell purifications were labeled by Cy3 (green), Cy5 (red), and Cy2 (blue) CyDye DIGE Fluor dyes, respectively. After quenching excessive CyDyes with lysine (10mM), labeled samples were merged. The merged sample contained PINCH-PrA and Flag-PINCH associated proteins as well as proteins pulled down in the control sample, each individually labeled with a fluorescent color (Green, Red, and Blue). The merged sample was then subjected to standard 2D-GE protocols. Briefly, the first dimension isoelectric focusing was performed using 11cm pH 5–8 IPG strip (ReadyStrip IPG, Bio-Rad) with Protean IEF Cell programmable power supply (Bio-Rad). In some experiments designed for mass spectrometric identification, 17 cm strips were used to maximize protein loading. DTT (50 mM) was the primary reducer during the isoelectric focusing. Second dimension SDS-PAGE was conducted with 10% polyacrylamide gels. The resulting gels were analyzed immediately by Typhoon Trio Laser scanner, employing the preset DIGE scanning protocol (GE Healthcare). Spot volumes in each color channel were quantified using plugins available for ImageJ (NIH), using Watershed plugin (available as free download at http://bigwww.epfl.ch/sage/soft/watershed/index.html) as previously described [40].

After determining spot locations in the multi-color/multi-sample 2D gel, we confirmed their reproducibility in 2D gels (n>3) containing the individual samples. Individual gels control for the possibility of cross-labeling between samples that may occur in iGEO tri-color labeling experiments due to insufficient quenching of excessive dyes. To adjust minor gel-to-gel variations in spot localization in individual gels, we used a 2D gel spot matching software (Progenesis SameSpots, Nonlinear Dynamics) that warp and deform individual gels to match up spot coordinates of multiple 2D gels. 2D gels of this stage were stained by a fluorescent protein dye (Deep Purple, GE Healthcare) based on the manufacturer’s protocol. We subjected spots from individual Deep Purple-stained gels to mass spectrometry for protein identification, to avoid any potential mass spectral artifacts from CyDye binding to lysine residues within the protein spots.

### Nano-LC-MS/MS analysis and Mascot database search

2D gels stained by Deep Purple were co-stained using an enhanced colloidal Coomassie blue staining protocol [41]. Spots of interest in colloidal blue-stained 2D gels were manually excised and subjected to in-gel tryptic digestion and mass spectrometric analysis. Detailed nano-LC-MS/MS and Mascot database search protocols are described in Additional file 8: Method S2.

## Abbreviations

iGEO: Interactions by Gel Electrophoresis Overlap; PINCH: Particularly Interesting New Cysteine and Histidine-rich protein; ILK: Integrin-Linked Kinase; RSU-1: Ras Suppressor-1; LIM Domain: a protein motif named after LIN-11, Isl1 & MEC-3; PrA: Protein A; MudPIT: Multi-dimensional Protein Identification Technology; 2D-GE: Two Dimensional Gel Electrophoresis; DIGE: Difference Gel Electrophoresis.

## Competing interests

The authors declare that they have no competing interests.

## Authors’ contributions

JLK and MY conceived and designed the experiments. MY and SMP performed the experiments. JLK and MY analyzed the data. JLK, SMP and MY contributed reagents, materials and analysis tools. JLK and MY drafted the manuscript. All authors read and approved the final manuscript.

## Supplementary Material

Additional file 1: Table S1 A complete list of proteins identified by MudPIT in replicate *Drosophila* embryo experiments (Embryo experiment 1). Click here for file

Additional file 2: Table S2 A complete list of proteins identified by MudPIT in replicate *Drosophila* S2 cell experiment. Click here for file

Additional file 3: Table S3 Candidate PINCH bcinding partners identified by MudPIT and present in both S2 cell and embryo experiments. Click here for file

Additional file 4: Figure S1 Source images of the tri-color labeled 2D gel shown in Figure 2B. By opening RGB-composite part (A) of this image using photo-editing software (*e.g.* Adobe Photoshop) and selecting color channel display, individual 2D gel spots can be inspected with respect to signal intensity for each color channel. Individual color channels are also displayed (B-D). Signal intensity of each spot was quantified using ImageJ plugins, using spot detection, volume calculation, and background subtraction (E). Click here for file

Additional file 5: Figure S2 Validation of ILK and RSU-1 by 2D immunoblotting (See Additional file 6: Method S1). **A**: Image of the protein gel from Flag-PINCH pull down (adapted from Figure 3E), showing spots of interest that were further analyzed by LC-MS/MS. Spots #54 and #113 were confirmed as ILK and RSU-1, respectively, by 2D immunoblotting, and therefore not submitted to LC-MS/MS analysis. Boxes highlighted in yellow and blue indicate the area in 2D protein gels where ILK or RSU-1 are anticipated to migrate. **B**: ILK signals on the 2D immunoblot within the area highlighted in yellow. The signal was concentrated at 50 kDa, the anticipatedsize of ILK. **C**: RSU-1 signals on the 2D immunoblot were detected as two major spots at 28 kDa, pl 6.7 (single head arrow) and 30 kDa, p; I 7.2. (double head arrow). The spot at 30 kDa, p I 7.2 matched with the location of spot #113 in 2D protein gels, and theoretical mass and pl of RSU-1. We therefore determined the spot #113 to be RSU-1. Click here for file

Additional file 6: Method S1 Two dimensional immunoblotting. Click here for file

Additional file 7 Mascot search results. Click here for file

Additional file 8: Method S2 Detailed procedures of LC-MS/MS. Click here for file
