# Supplementary figures and images for: Interactions by 2D Gel Electrophoresis Overlap (iGEO): a novel high fidelity approach to identify constituents of protein complexes
Source: Proteome Sci. 2013 May 12;11:21. doi: 10.1186/1477-5956-11-21 (PMC3688448; doi:10.1186/1477-5956-11-21)

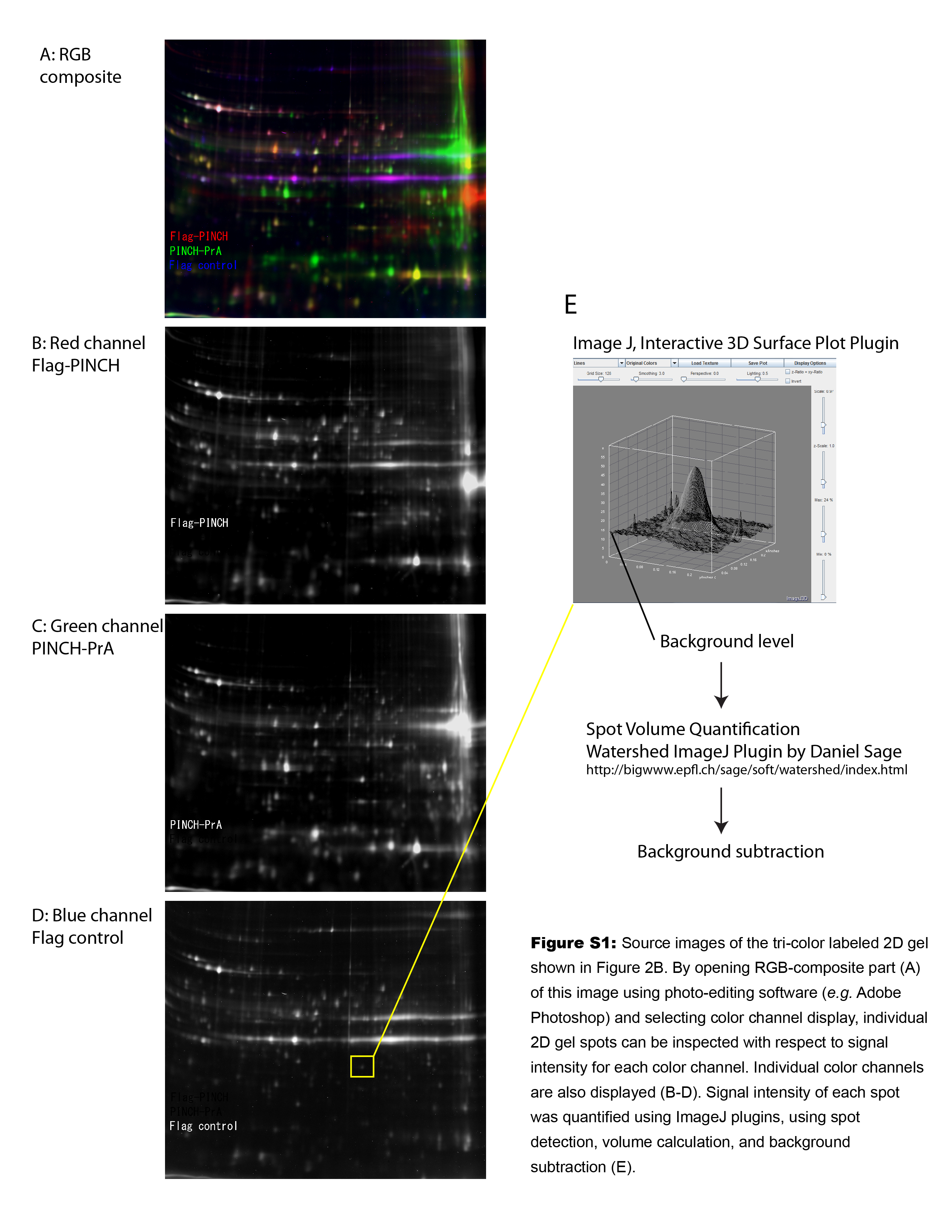

Supplement: Additional file 4: Figure S1 — Source images of the tri-color labeled 2D gel shown in Figure 2B. By opening RGB-composite part (A) of this image using photo-editing software (e.g. Adobe Photoshop) and selecting color channel display, individual 2D gel spots can be inspected with respect to signal intensity for each color channel. Individual color channels are also displayed (B-D). Signal intensity of each spot was quantified using ImageJ plugins, using spot detection, volume calculation, and background subtraction (E). [file 1477-5956-11-21-S4.jpg]

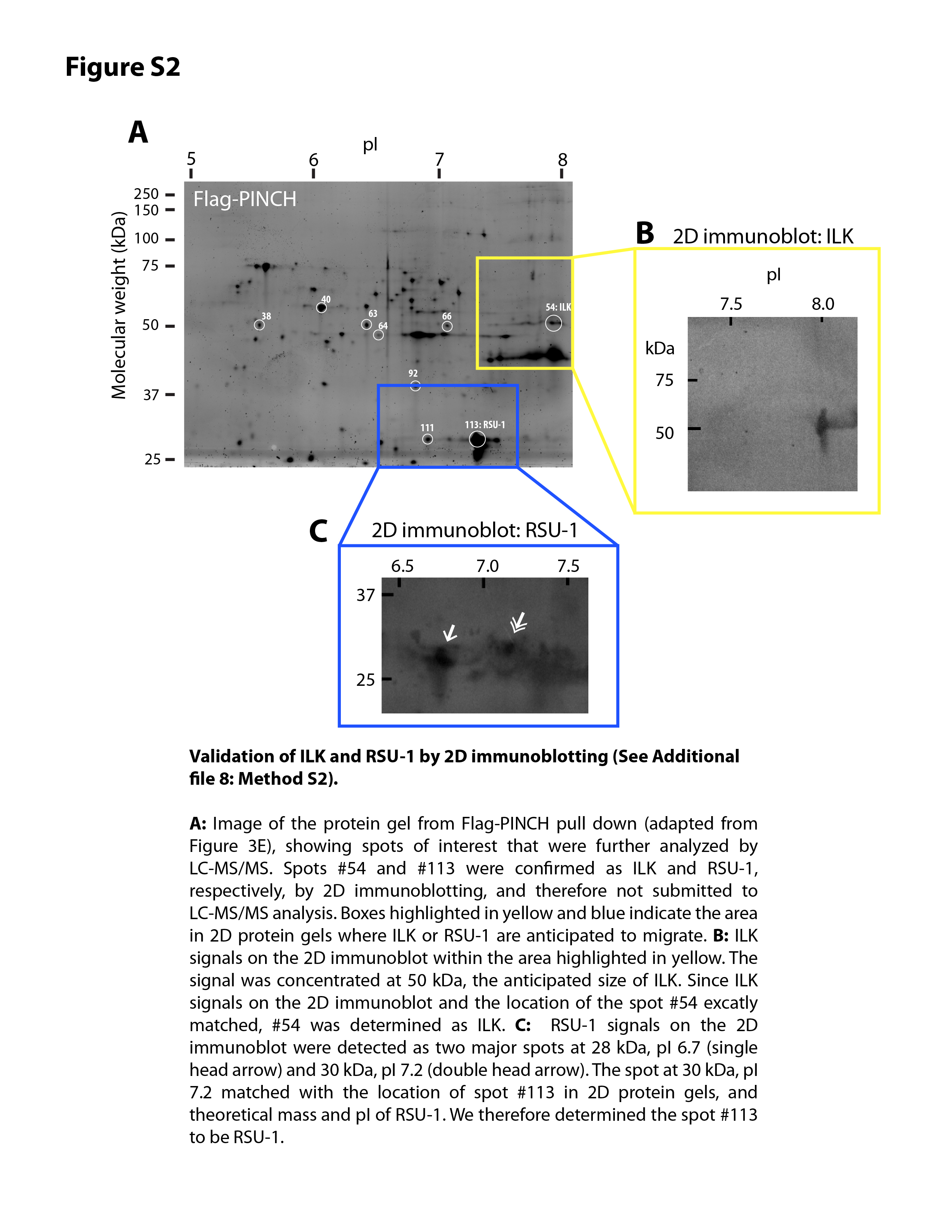

Supplement: Additional file 5: Figure S2 — Validation of ILK and RSU-1 by 2D immunoblotting (See Additional file 6: Method S1). A: Image of the protein gel from Flag-PINCH pull down (adapted from Figure 3E), showing spots of interest that were further analyzed by LC-MS/MS. Spots #54 and #113 were confirmed as ILK and RSU-1, respectively, by 2D immunoblotting, and therefore not submitted to LC-MS/MS analysis. Boxes highlighted in yellow and blue indicate the area in 2D protein gels where ILK or RSU-1 are anticipated to migrate. B: ILK signals on the 2D immunoblot within the area highlighted in yellow. The signal was concentrated at 50 kDa, the anticipatedsize of ILK. C: RSU-1 signals on the 2D immunoblot were detected as two major spots at 28 kDa, pl 6.7 (single head arrow) and 30 kDa, p; I 7.2. (double head arrow). The spot at 30 kDa, p I 7.2 matched with the location of spot #113 in 2D protein gels, and theoretical mass and pl of RSU-1. We therefore determined the spot #113 to be RSU-1. [file 1477-5956-11-21-S5.jpg]
