# Supplementary material for: Interactions by 2D Gel Electrophoresis Overlap (iGEO): a novel high fidelity approach to identify constituents of protein complexes
Source: Proteome Sci. 2013 May 12;11:21. doi: 10.1186/1477-5956-11-21 (PMC3688448; doi:10.1186/1477-5956-11-21)
Supplement: Additional file 6: Method S1 — Two dimensional immunoblotting. [file 1477-5956-11-21-S6.docx]

**Method S1: Two dimensional immunoblotting**

Pull down samples from S2 cells expressing Flag-PINCH or PINCH-PrA were resolved by 2D-GE as outlined in the Methods section. Generally, protein loading was reduced 75-90% as compared to protein gels so that western blotting signal does not produce extreme background or over-saturated signal. Following the second dimension SDS-PAGE, proteins were electro-blotted to PVDF membranes overnight. Molecular weight markers and pI landmarks (edges of the IPG strip) were marked on the PVDF membrane. Blotted membranes were blocked in 3% BSA, followed by incubation with anti-ILK (BD Transduction Laboratory) or anti-RSU-1[6] antibody, respectively. Signals were detected by HRP-conjugated secondary antibodies followed by enhanced chemiluminescence.
