# Supplementary material for: Interactions by 2D Gel Electrophoresis Overlap (iGEO): a novel high fidelity approach to identify constituents of protein complexes
Source: Proteome Sci. 2013 May 12;11:21. doi: 10.1186/1477-5956-11-21 (PMC3688448; doi:10.1186/1477-5956-11-21)
Supplement: Additional file 7 — Mascot search results. [file 1477-5956-11-21-S7.pdf]

MATRIX SCIENCE Mascot Search Results

User	:	Julie Kadrmas; gel band: #38
Email	:	110223FT_JulieKadrmas_38
Search title	:	NCBI Drosophila DB trypsin specific search: Oxidation (MHW)
MS data file	:	C:\Documents and Settings\cnelson\Desktop\Merge Files_2011\February_2011\merge_110223FT_JulieKad
Database	:	NCBInr (5335292 sequences; 1845185051 residues)
Taxonomy	:	Drosophila (fruit flies) (50248 sequences)
Timestamp	:	24 Feb 2011 at 19:10:11 GMT
Protein hits	:	gi|18860063 Rpt3 CG16916-PA [Drosophila melanogaster]
		gi|3426089 polyprotein [Drosophila melanogaster]
		gi|11323019 pol [Drosophila melanogaster]

Probability Based Mowse Score

Ions score is -10*Log(P), where P is the probability that the observed match is a random event.
Individual ions scores > 23 indicate identity or extensive homology (p<0.05).
Protein scores are derived from ions scores as a non-probabilistic basis for ranking protein hits.

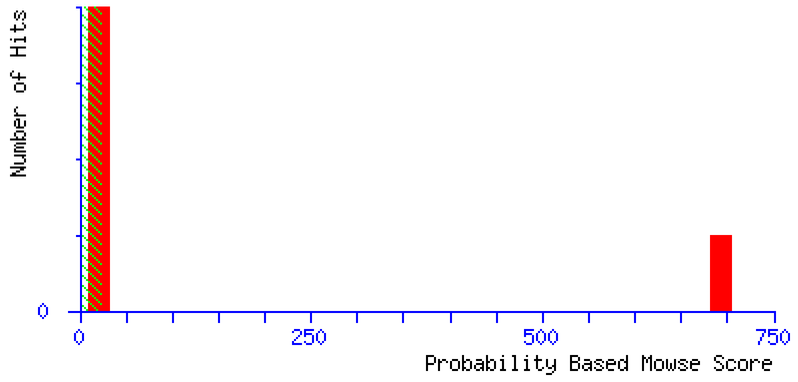

Peptide Summary Report

Format As | Peptide Summary | Help

Significance threshold p< 0.05 Max. number of hits AUTO

Standard scoring (•) MudPIT scoring ( ) Ions score or expect cut-off [20] Show sub-sets [0]

Show pop-ups ( ) Suppress pop-ups (•) Sort unassigned [Decreasing Score] Require bold red [x]

[Select All] [Select None] [Search Selected] [ ] **Error tolerant** [Archive Report]

1. gi|18860063 **Mass:** 46969 **Score:** 693 **Queries matched:** 24 **emPAI:** 3.77

Rpt3 CG16916-PA [Drosophila melanogaster]

[ ] Check to include this hit in error tolerant search or archive report

	Query	Observed	Mr(expt)	Mr(calc)	ppm	Miss	Score	Expect	Rank	Peptide
[x]	1366	706.4517	705.4444	705.4425	2.68	0	21	0.03	1	R.YIVLAK.D
[x]	1471	396.2069	790.3993	790.3973	2.47	0	21	0.18	1	K.YLGEGPR.M
[x]	1600	469.7492	937.4838	937.4804	3.62	1	26	0.028	1	R.MVRDVFR.L + Oxidation (M)
[x]	1621	496.7754	991.5362	991.5339	2.35	0	59	1.1e-005	1	R.VVGSEFVQK.Y
[x]	1624	995.5785	994.5712	994.5699	1.29	0	(30)	0.0089	1	R.LVFSTITSK.M
[x]	1625	498.2930	994.5714	994.5699	1.52	0	45	0.00025	1	R.LVFSTITSK.M
[x]	1663	576.3441	1150.6737	1150.6710	2.38	1	36	0.0012	1	K.RLVFSTITSK.M
[x]	1672	613.3465	1224.6785	1224.6754	2.53	1	49	9.8e-005	1	R.YIVLAKDFEK.G
[x]	1673	409.2339	1224.6798	1224.6754	3.62	1	(23)	0.028	1	R.YIVLAKDFEK.G
[x]	1681	424.2481	1269.7223	1269.7193	2.36	2	31	0.0056	1	R.KIEFPLPDRR.Q
[x]	1686	437.5707	1309.6902	1309.6891	0.83	0	(37)	0.0028	1	K.AVAHHTTASFIR.V
[x]	1687	655.8548	1309.6951	1309.6891	4.57	0	49	0.00016	1	K.AVAHHTTASFIR.V
[x]	1694	701.3615	1400.7084	1400.7048	2.59	1	(35)	0.004	1	K.EYLHAQEEVKR.I
[x]	1695	467.9104	1400.7095	1400.7048	3.37	1	44	0.00059	1	K.EYLHAQEEVKR.I
[x]	1698	498.2438	1491.7097	1491.7066	2.05	1	36	0.0029	1	R.FDAQTGADREVQR.I
[x]	1699	746.8630	1491.7114	1491.7066	3.21	1	(26)	0.03	1	R.FDAQTGADREVQR.I
[x]	1705	824.9144	1647.8142	1647.8077	3.96	2	31	0.016	1	K.RFDAQTGADREVQR.I
[x]	1711	844.9510	1687.8874	1687.8821	3.12	0	46	0.00042	1	R.EAVELPLTHFELYK.Q
[x]	1739	675.3043	2022.8910	2022.8840	3.46	0	22	0.032	1	K.DAHSSLDELDMEDLYVR.Y + Oxidation (M
[x]	1743	708.3394	2121.9963	2121.9888	3.54	0	29	0.016	1	K.MNLSEDVDLEEFVARPDK.I + Oxidation (
[x]	1748	1085.0417	2168.0688	2168.0637	2.34	1	76	3.7e-007	1	K.TLEFIEVQEEYIKDEQR.N
[x]	1749	723.6969	2168.0690	2168.0637	2.43	1	(47)	0.00034	1	K.TLEFIEVQEEYIKDEQR.N
	1752	1104.5680	2207.1214	2207.1144	3.17	0	71	1.5e-006	1	R.ILLELLNQMDGFDQTTNVK.V + Oxidation

☑										
☑	1753	736.7148	2207.1225	2207.1144	3.69	0	(61)	1.5e-005	1	R.ILLELLNQMDGFDQTTNVK.V + Oxidation

---

2. gi\|3426089 **Mass:** 177601 **Score:** 31 **Queries matched:** 5 **emPAI:** 0.02
polyprotein [Drosophila melanogaster]

☐ Check to include this hit in error tolerant search or archive report

	Query	Observed	Mr(expt)	Mr(calc)	ppm	Miss	Score	Expect	Rank	Peptide
☑	1295	672.4048	671.3975	671.3966	1.27	0	(27)	0.028	1	K.TVSLPR.L
☑	1296	672.4049	671.3976	671.3966	1.42	0	31	0.01	1	K.TVSLPR.L
☑	1297	672.4050	671.3977	671.3966	1.60	0	(21)	0.12	1	K.TVSLPR.L
☑	1298	672.4052	671.3979	671.3966	1.95	0	(23)	0.077	1	K.TVSLPR.L
☑	1299	672.4055	671.3982	671.3966	2.37	0	(27)	0.027	1	K.TVSLPR.L

Proteins matching the same set of peptides:
gi\|21214752 **Mass:** 206214 **Score:** 31 **Queries matched:** 5
gag-pol polyprotein precursor; hypothetical protein [Drosophila melanogaster]
gi\|25009978 **Mass:** 83774 **Score:** 31 **Queries matched:** 5
GH06606p [Drosophila melanogaster]

---

3. gi\|11323019 **Mass:** 139125 **Score:** 23 **Queries matched:** 1
pol [Drosophila melanogaster]

☐ Check to include this hit in error tolerant search or archive report

	Query	Observed	Mr(expt)	Mr(calc)	ppm	Miss	Score	Expect	Rank	Peptide
☑	1520	423.2277	844.4409	844.4443	-4.06	0	23	0.082	1	K.SPALWGSK.Q

Peptide matches not assigned to protein hits: (no details means no match)

	Query	Observed	Mr(expt)	Mr(calc)	ppm	Miss	Score	Expect	Rank	Peptide
☑	1473	791.4439	790.4366	790.4371	-0.65	1	19	0.33	1	QMLKQK + Oxidation (M)
☑	1525	423.7204	845.4263	845.4243	2.42	0	19	0.22	1	ALAEAESR
☑	1594	466.7462	931.4778	931.4770	0.82	2	19	0.16	1	ACSPRRSR

Mascot Search Results

User : **Julie Kadrmas; gel band: #40**
Email : **110223FT_JulieKadrmas_40**
Search title : **NCBI Drosophila DB trypsin specific search: Oxidation (MHW)**
MS data file : **C:\Documents and Settings\cnelson\Desktop\Merge Files_2011\February_2011\merge_110223FT_JulieKad**
Database : **NCBInr (5335292 sequences; 1845185051 residues)**
Taxonomy : **Drosophila (fruit flies) (50248 sequences)**
Timestamp : **24 Feb 2011 at 19:12:43 GMT**
Protein hits : gi|45551086 ERp60 CG8983-PA, isoform A [Drosophila melanogaster]
gi|157570 gamma-tubulin
gi|158739 beta-1 tubulin
gi|20129399 Aldehyde dehydrogenase CG3752-PA [Drosophila melanogaster]
gi|6942155 putative cytoplasmic aminopeptidase [Drosophila melanogaster]
gi|1923274 Tpr homolog [Drosophila melanogaster]
gi|17944482 RH04336p [Drosophila melanogaster]
gi|17945899 RE40293p [Drosophila melanogaster]
gi|3426089 polyprotein [Drosophila melanogaster]
gi|304728 transcription factor
gi|19920576 CG3557-PA [Drosophila melanogaster]
gi|21429138 RE42508p [Drosophila melanogaster]
gi|9799628 Nut2 [Drosophila melanogaster]
gi|45550970 CG5792-PB, isoform B [Drosophila melanogaster]
gi|24644950 CG2767-PA [Drosophila melanogaster]

Probability Based Mowse Score

Ions score is -10*Log(P), where P is the probability that the observed match is a random event.
Individual ions scores > 24 indicate identity or extensive homology (p<0.05).
Protein scores are derived from ions scores as a non-probabilistic basis for ranking protein hits.

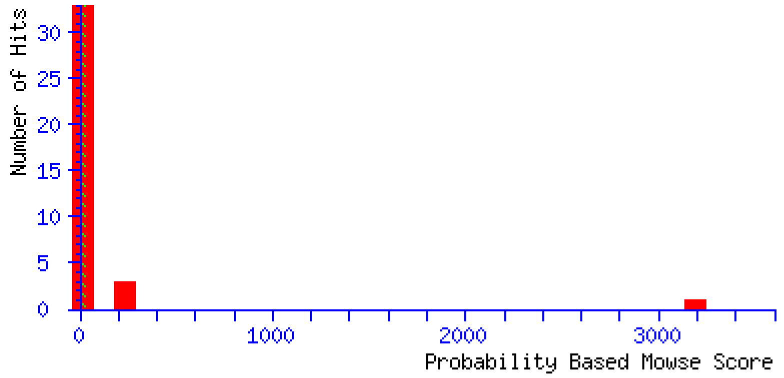

Peptide Summary Report

Format As | Peptide Summary | Help

Significance threshold p< 0.05 | Max. number of hits AUTO

Standard scoring ◉ MudPIT scoring ○ | Ions score or expect cut-off 20 | Show sub-sets 0

Show pop-ups ○ Suppress pop-ups ◉ | Sort unassigned Decreasing Score | Require bold red ☑

Select All | Select None | Search Selected | ☐ **Error tolerant** | Archive Report

1. gi|45551086 **Mass:** 55336 **Score:** 3195 **Queries matched:** 146 **emPAI:** 159.92
ERp60 CG8983-PA, isoform A [Drosophila melanogaster]
☐ Check to include this hit in error tolerant search or archive report

	Query	Observed	Mr(expt)	Mr(calc)	ppm	Miss	Score	Expect	Rank	Peptide
☑	636	487.3243	486.3170	486.3166	0.88	0	29	0.062	1	K.VAVAK.N
☑	637	487.3243	486.3170	486.3166	0.88	0	(24)	0.21	1	K.VAVAK.N
☑	638	487.3243	486.3171	486.3166	1.01	0	(29)	0.063	1	K.VAVAK.N
☑	1105	603.3359	602.3286	602.3275	1.78	0	30	0.03	1	K.EVLDK.Q
☑	1106	603.3367	602.3295	602.3275	3.19	0	(23)	0.17	1	K.EVLDK.Q
☑	1146	613.4402	612.4330	612.4323	1.16	0	27	0.022	1	K.IVLIR.A
	1147	613.4403	612.4331	612.4323	1.30	0	(23)	0.049	1	K.IVLIR.A

☑										
☑	1148	613.4406	612.4333	612.4323	1.65	0	(26)	0.03	1	K.IVLIR.A
☑	1187	623.3413	622.3340	622.3326	2.28	0	28	0.026	1	K.FLDTK.D
	1291	660.3943	659.3870	659.3854	2.53	0	(31)	0.013	4	R.TVAELK.K
	1292	660.3946	659.3874	659.3854	3.03	0	32	0.011	4	R.TVAELK.K
☑	1338	675.3682	674.3610	674.3599	1.59	0	(28)	0.039	1	R.EASGIAK.Y
☑	1339	675.3683	674.3610	674.3599	1.68	0	(26)	0.061	1	R.EASGIAK.Y
☑	1340	675.3689	674.3617	674.3599	2.63	0	(25)	0.1	1	R.EASGIAK.Y
☑	1341	675.3690	674.3617	674.3599	2.72	0	29	0.035	1	R.EASGIAK.Y
☑	1482	746.4059	745.3987	745.3970	2.25	1	29	0.03	1	R.DEKNLK.Y
☑	1503	379.2143	756.4140	756.4130	1.37	0	(36)	0.0025	1	R.AQVGPASK.T
☑	1504	379.2143	756.4141	756.4130	1.45	0	51	9.6e-005	1	R.AQVGPASK.T
☑	1505	757.4215	756.4142	756.4130	1.56	0	(21)	0.08	1	R.AQVGPASK.T
☑	1506	757.4215	756.4142	756.4130	1.64	0	(34)	0.0042	1	R.AQVGPASK.T
☑	1507	379.2145	756.4145	756.4130	2.02	0	(48)	0.00018	1	R.AQVGPASK.T
☑	1508	757.4221	756.4148	756.4130	2.37	0	(23)	0.05	1	R.AQVGPASK.T
☑	1524	383.7147	765.4149	765.4133	2.10	0	(26)	0.019	1	R.APHLSNK.F
☑	1525	383.7148	765.4150	765.4133	2.14	0	34	0.0031	1	R.APHLSNK.F
☑	1526	766.4226	765.4153	765.4133	2.64	0	(24)	0.03	1	R.APHLSNK.F
☑	1555	394.7482	787.4818	787.4803	1.85	1	29	0.014	1	R.TVAELKK.F
	1556	394.7483	787.4820	787.4803	2.09	1	(20)	0.12	2	R.TVAELKK.F
☑	1558	394.7484	787.4823	787.4803	2.54	1	(28)	0.019	1	R.TVAELKK.F
☑	1559	394.7485	787.4825	787.4803	2.70	1	(28)	0.017	1	R.TVAELKK.F
☑	1560	788.4915	787.4842	787.4803	4.89	1	(21)	0.085	1	R.TVAELKK.F
☑	1579	791.4162	790.4089	790.4072	2.12	0	(29)	0.02	1	K.EATTELK.G
☑	1580	791.4162	790.4090	790.4072	2.18	0	(21)	0.15	1	K.EATTELK.G
☑	1581	396.2118	790.4091	790.4072	2.36	0	31	0.013	1	K.EATTELK.G
☑	1586	398.6911	795.3676	795.3664	1.57	0	25	0.021	1	K.GTNYWR.N
☑	1589	399.2061	796.3976	796.3967	1.23	0	25	0.02	1	K.FESSSIK.F
☑	1593	799.3848	798.3775	798.3759	1.94	0	31	0.0036	1	K.DDDPPIK.L
☑	1674	424.7482	847.4818	847.4803	1.80	0	28	0.015	1	R.LKPEYAK.A
☑	1725	433.2195	864.4244	864.4229	1.69	0	36	0.0035	1	R.EVDDFLK.Y
☑	1871	502.7991	1003.5837	1003.5814	2.25	1	(27)	0.017	1	K.RLKPEYAK.A
	1872	502.7998	1003.5851	1003.5814	3.68	1	33	0.0046	1	K.RLKPEYAK.A

☑										
☑	1968	557.7924	1113.5702	1113.5706	-0.41	0	(29)	0.016	1	K.YSVSGYPTLK.I
☑	1969	1114.5789	1113.5716	1113.5706	0.87	0	(32)	0.0082	1	K.YSVSGYPTLK.I
☑	1970	557.7931	1113.5717	1113.5706	0.97	0	50	0.00014	1	K.YSVSGYPTLK.I
☑	1971	557.7944	1113.5743	1113.5706	3.27	0	(39)	0.0016	1	K.YSVSGYPTLK.I
☑	1972	557.7946	1113.5747	1113.5706	3.65	0	(28)	0.02	1	K.YSVSGYPTLK.I
☑	1982	565.3100	1128.6054	1128.6026	2.48	0	55	4.9e-005	1	K.LQDEDVAIVK.M
☑	1988	571.2880	1140.5615	1140.5597	1.57	1	32	0.0074	1	R.EASGIAKYMR.A + Oxidation (M)
☑	2007	389.2011	1164.5813	1164.5788	2.13	0	(21)	0.1	1	K.ENFHGLVGHR.T
☑	2008	583.2981	1164.5816	1164.5788	2.35	0	49	0.00019	1	K.ENFHGLVGHR.T
☑	2032	603.3402	1204.6658	1204.6645	1.10	0	(35)	0.0045	1	R.GFPTLFWLPK.D
☑	2034	603.3405	1204.6665	1204.6645	1.66	0	36	0.0029	1	R.GFPTLFWLPK.D
☑	2044	611.3376	1220.6607	1220.6594	1.08	0	(27)	0.034	1	R.GFPTLFWLPK.D + Oxidation (HW)
☑	2045	611.3381	1220.6617	1220.6594	1.90	0	(26)	0.034	1	R.GFPTLFWLPK.D + Oxidation (HW)
☑	2046	611.3383	1220.6620	1220.6594	2.11	0	(26)	0.034	1	R.GFPTLFWLPK.D + Oxidation (HW)
☑	2080	422.8834	1265.6283	1265.6252	2.48	1	(31)	0.019	1	K.EATTELKGFDR.S
☑	2081	633.8216	1265.6287	1265.6252	2.79	1	47	0.00044	1	K.EATTELKGFDR.S
☑	2087	424.5831	1270.7273	1270.7245	2.24	1	(37)	0.0019	1	K.QGETDKIVLIR.A
☑	2088	636.3712	1270.7279	1270.7245	2.72	1	51	7.8e-005	1	K.QGETDKIVLIR.A
☑	2093	646.3444	1290.6742	1290.6707	2.70	0	53	8.9e-005	1	K.LSPIYEELAEK.L
☑	2099	651.8699	1301.7253	1301.7231	1.69	0	59	2e-005	1	K.LLANELEPYIK.S
☑	2100	651.8703	1301.7260	1301.7231	2.25	0	(47)	0.00025	1	K.LLANELEPYIK.S
☑	2101	1302.7347	1301.7275	1301.7231	3.39	0	(30)	0.0082	1	K.LLANELEPYIK.S
☑	2127	670.8601	1339.7057	1339.7023	2.52	1	51	0.00012	1	R.EVDDFLKYIAK.E
☑	2131	459.2345	1374.6818	1374.6779	2.78	1	(37)	0.0029	1	R.FGHSSEKEVLDK.Q
☑	2132	688.3488	1374.6831	1374.6779	3.75	1	56	4.6e-005	1	R.FGHSSEKEVLDK.Q
☑	2159	704.3120	1406.6095	1406.6062	2.35	0	(75)	1.5e-007	1	R.QDEVSQDYNGPR.E
☑	2160	704.3121	1406.6097	1406.6062	2.43	0	(77)	8.6e-008	1	R.QDEVSQDYNGPR.E
☑	2161	704.3125	1406.6105	1406.6062	3.04	0	81	3.7e-008	1	R.QDEVSQDYNGPR.E
☑	2168	470.9217	1409.7434	1409.7402	2.27	1	(34)	0.0047	1	K.AAEIVKDDDPPIK.L
☑	2169	470.9218	1409.7434	1409.7402	2.30	1	(33)	0.0053	1	K.AAEIVKDDDPPIK.L
☑	2170	705.8790	1409.7434	1409.7402	2.31	1	(35)	0.0034	1	K.AAEIVKDDDPPIK.L
☑	2171	705.8794	1409.7443	1409.7402	2.91	1	62	6.9e-006	1	K.AAEIVKDDDPPIK.L

☑	2175	710.3921	1418.7696	1418.7656	2.77	1	64	4.9e-006	1	K.KLSPIYEELAEK.L
☑	2178	712.3850	1422.7554	1422.7507	3.30	0	(85)	3.3e-008	1	K.EFVGQINFAIASK.D
☑	2179	712.3852	1422.7559	1422.7507	3.65	0	90	1.2e-008	1	K.EFVGQINFAIASK.D
☑	2230	760.4210	1518.8274	1518.8235	2.55	1	45	0.00039	1	R.GFPTLFWLPKDAK.N
☑	2235	766.8612	1531.7078	1531.7042	2.34	0	94	3.4e-009	1	K.FEGSSESDLSTFVK.E
☑	2236	766.8620	1531.7095	1531.7042	3.45	0	(72)	4.9e-007	1	K.FEGSSESDLSTFVK.E
☑	2237	766.8624	1531.7102	1531.7042	3.93	0	(50)	8.3e-005	1	K.FEGSSESDLSTFVK.E
☑	2243	515.6085	1543.8036	1543.7994	2.67	1	(21)	0.11	1	R.APHLSNKFESSSIK.F
☑	2244	772.9107	1543.8069	1543.7994	4.82	1	49	0.00022	1	R.APHLSNKFESSSIK.F
☑	2263	791.8500	1581.6854	1581.6835	1.20	0	(38)	0.00053	1	K.DTTLFGYFSDSDSK.L
☑	2264	791.8512	1581.6878	1581.6835	2.75	0	55	2.2e-005	1	K.DTTLFGYFSDSDSK.L
☑	2265	791.8518	1581.6890	1581.6835	3.48	0	(29)	0.01	1	K.DTTLFGYFSDSDSK.L
☑	2266	791.8523	1581.6901	1581.6835	4.17	0	(42)	0.00051	1	K.DTTLFGYFSDSDSK.L
☑	2298	559.2640	1674.7701	1674.7672	1.75	0	(39)	0.0013	1	K.MDATANDVPPEFNVR.G
☑	2299	838.3934	1674.7723	1674.7672	3.04	0	(40)	0.0011	1	K.MDATANDVPPEFNVR.G
☑	2300	838.3936	1674.7726	1674.7672	3.25	0	(57)	1.9e-005	1	K.MDATANDVPPEFNVR.G
☑	2301	838.3936	1674.7727	1674.7672	3.29	0	(82)	5.9e-008	1	K.MDATANDVPPEFNVR.G
☑	2309	846.3901	1690.7656	1690.7621	2.08	0	102	5.6e-010	1	K.MDATANDVPPEFNVR.G + Oxidation (M)
☑	2310	846.3905	1690.7665	1690.7621	2.58	0	(60)	8.1e-006	1	K.MDATANDVPPEFNVR.G + Oxidation (M)
☑	2311	846.3907	1690.7668	1690.7621	2.80	0	(59)	1e-005	1	K.MDATANDVPPEFNVR.G + Oxidation (M)
☑	2312	564.5964	1690.7673	1690.7621	3.08	0	(36)	0.0021	1	K.MDATANDVPPEFNVR.G + Oxidation (M)
☑	2313	846.3911	1690.7677	1690.7621	3.31	0	(81)	7.2e-008	1	K.MDATANDVPPEFNVR.G + Oxidation (M)
☑	2319	565.6227	1693.8464	1693.8424	2.38	2	35	0.0045	1	K.YRFGHSSEKEVLDK.Q
☑	2326	849.8990	1697.7834	1697.7784	2.90	0	61	9.7e-006	1	K.DEFSVENLQDFVEK.L
☑	2342	861.4851	1720.9557	1720.9512	2.64	1	45	0.00028	1	K.VAKEFVGQINFAIASK.D
☑	2343	574.6594	1720.9564	1720.9512	3.00	1	(40)	0.00071	1	K.VAKEFVGQINFAIASK.D
☑	2344	861.9870	1721.9595	1721.9563	1.85	2	(36)	0.0015	1	K.AAEIVKDDDPPIKLAK.V
☑	2345	574.9941	1721.9603	1721.9563	2.33	2	(47)	0.00013	1	K.AAEIVKDDDPPIKLAK.V
☑	2346	574.9941	1721.9605	1721.9563	2.43	2	(45)	0.00023	1	K.AAEIVKDDDPPIKLAK.V
☑	2347	861.9884	1721.9622	1721.9563	3.41	2	55	2.1e-005	1	K.AAEIVKDDDPPIKLAK.V
☑	2348	581.3102	1740.9087	1740.9046	2.35	2	(46)	0.00038	1	K.YIAKEATTELKGFDR.S
☑	2349	581.3104	1740.9094	1740.9046	2.77	2	54	5e-005	1	K.YIAKEATTELKGFDR.S
☑	2367	608.6283	1822.8631	1822.8598	1.78	1	(38)	0.0021	1	K.IFRQDEVSQDYNGPR.E

☑	2368	608.6288	1822.8647	1822.8598	2.68	1	(41)	0.0011	1	K.IFRQDEVSQDYNGPR.E
☑	2369	912.4398	1822.8651	1822.8598	2.92	1	(61)	1.1e-005	1	K.IFRQDEVSQDYNGPR.E
☑	2370	912.4403	1822.8660	1822.8598	3.39	1	63	6.5e-006	1	K.IFRQDEVSQDYNGPR.E
☑	2386	617.6690	1849.9851	1849.9785	3.56	1	(33)	0.0076	1	K.SEPIPESNDAPVKVAVAK.N
☑	2387	925.9999	1849.9853	1849.9785	3.65	1	88	2.6e-008	1	K.SEPIPESNDAPVKVAVAK.N
☑	2392	619.3565	1855.0477	1855.0415	3.36	2	50	5.7e-005	1	K.EVLDKQGETDKIVLIR.A
☑	2398	947.9589	1893.9032	1893.8996	1.87	1	64	8.1e-006	1	K.DTTLFGYFSDSDSKLAK.I
☑	2399	632.3086	1893.9040	1893.8996	2.28	1	(28)	0.032	1	K.DTTLFGYFSDSDSKLAK.I
☑	2440	678.6660	2032.9760	2032.9702	2.89	2	39	0.002	1	R.FGHSSEKEVLDKQGETDK.I
☑	2444	688.6591	2062.9554	2062.9556	-0.06	1	33	0.0071	1	R.QDEVSQDYNGPREASGIAK.Y
☑	2445	1033.0011	2063.9877	2063.9840	1.79	0	(89)	2e-008	1	K.DFQNPLITAYYSVDYQK.N
☑	2446	1033.0017	2063.9889	2063.9840	2.38	0	95	4.7e-009	1	K.DFQNPLITAYYSVDYQK.N
☑	2447	689.0041	2063.9905	2063.9840	3.16	0	(64)	5.8e-006	1	K.DFQNPLITAYYSVDYQK.N
☑	2457	725.3622	2173.0647	2173.0579	3.14	1	(50)	0.00019	1	K.YALKDEFSVENLQDFVEK.L
☑	2458	1087.5414	2173.0682	2173.0579	4.72	1	85	5.8e-008	1	K.YALKDEFSVENLQDFVEK.L
☑	2459	729.6779	2186.0119	2186.0056	2.91	1	(46)	0.00038	1	K.FLDTKDTTLFGYFSDSDSK.L
☑	2460	1094.0135	2186.0125	2186.0056	3.16	1	106	3.4e-010	1	K.FLDTKDTTLFGYFSDSDSK.L
☑	2461	729.6788	2186.0145	2186.0056	4.08	1	(21)	0.12	1	K.FLDTKDTTLFGYFSDSDSK.L
☑	2496	771.0404	2310.0994	2310.0903	3.92	1	47	0.00033	1	K.FESSSIKFEGSSESDLSTFVK.E
☑	2497	771.0408	2310.1007	2310.0903	4.48	1	(41)	0.00094	1	K.FESSSIKFEGSSESDLSTFVK.E
☑	2499	772.3745	2314.1015	2314.1005	0.43	2	34	0.0073	1	K.KFLDTKDTTLFGYFSDSDSK.L
☑	2504	801.4305	2401.2696	2401.2628	2.85	1	(50)	8.8e-005	1	K.LSPIYEELAEKLQDEDVAIVK.M
☑	2505	1201.6422	2401.2699	2401.2628	2.98	1	65	2.7e-006	1	K.LSPIYEELAEKLQDEDVAIVK.M
☑	2506	1202.5951	2403.1757	2403.1747	0.43	1	47	0.00039	1	K.DFQNPLITAYYSVDYQKNPK.G
☑	2507	1202.5988	2403.1830	2403.1747	3.48	1	(35)	0.0058	1	K.DFQNPLITAYYSVDYQKNPK.G
☑	2508	802.0683	2403.1831	2403.1747	3.50	1	(37)	0.0041	1	K.DFQNPLITAYYSVDYQKNPK.G
☑	2509	802.0685	2403.1836	2403.1747	3.73	1	(28)	0.029	1	K.DFQNPLITAYYSVDYQKNPK.G
☑	2513	827.4123	2479.2151	2479.2091	2.42	2	34	0.0079	1	K.IFRQDEVSQDYNGPREASGIAK.Y
☑	2516	1250.1217	2498.2289	2498.2217	2.89	2	(50)	0.00017	1	K.FLDTKDTTLFGYFSDSDSKLAK.I
☑	2517	833.7509	2498.2307	2498.2217	3.62	2	56	5.2e-005	1	K.FLDTKDTTLFGYFSDSDSKLAK.I
☑	2518	844.1299	2529.3680	2529.3577	4.06	2	55	3.1e-005	1	K.KLSPIYEELAEKLQDEDVAIVK.M
☑	2524	889.4711	2665.3916	2665.3850	2.46	1	(36)	0.0038	1	K.LLANELEPYIKSEPIPESNDAPVK.V
☑	2525	889.4717	2665.3933	2665.3850	3.12	1	39	0.0016	1	K.LLANELEPYIKSEPIPESNDAPVK.V

2534	908.4475	2722.3207	2722.3126	2.97	1	(42)	0.0011	1	R.TQDSVKDFQNPLITAYYSVDYQK.N
2535	1362.1676	2722.3207	2722.3126	2.97	1	50	0.00018	1	R.TQDSVKDFQNPLITAYYSVDYQK.N
2536	908.4482	2722.3226	2722.3126	3.67	1	(48)	0.00025	1	R.TQDSVKDFQNPLITAYYSVDYQK.N
2540	934.7944	2801.3613	2801.3542	2.54	1	(41)	0.0014	1	K.LQDEDVAIVKMDATANDVPPEFNVR.G + Oxid
2541	934.7952	2801.3637	2801.3542	3.39	1	44	0.00068	1	K.LQDEDVAIVKMDATANDVPPEFNVR.G + Oxid
2542	934.7963	2801.3672	2801.3542	4.63	1	(25)	0.059	1	K.LQDEDVAIVKMDATANDVPPEFNVR.G + Oxid
2545	1021.5118	3061.5135	3061.5033	3.35	2	83	1.1e-007	1	R.TQDSVKDFQNPLITAYYSVDYQKNPK.G
2546	1045.5743	3133.7010	3133.6910	3.19	2	59	5.3e-006	1	K.LLANELEPYIKSEPIPESNDAPVKVAVAK.N

---

2. gi|157570 **Mass:** 53278 **Score:** 244 **Queries matched:** 5 **emPAI:** 0.27

gamma-tubulin

☐ Check to include this hit in error tolerant search or archive report

Query	Observed	Mr(expt)	Mr(calc)	ppm	Miss	Score	Expect	Rank	Peptide
1894	513.3047	1024.5949	1024.5917	3.13	0	37	0.00093	1	R.AVLLDLEPR.V
1895	513.3051	1024.5956	1024.5917	3.84	0	(32)	0.0034	1	R.AVLLDLEPR.V
2118	664.3521	1326.6896	1326.6853	3.23	0	71	7.5e-007	1	R.VINTIMGSVYSK.L + Oxidation (M)
2122	670.3494	1338.6842	1338.6819	1.68	0	45	0.00067	1	K.LYNPENVYLSK.H
2395	937.0079	1872.0013	1871.9953	3.20	0	90	1.3e-008	1	R.LTTAADSVVVLDNTALNR.I

Proteins matching the same set of peptides:

gi|3342456 **Mass:** 37046 **Score:** 244 **Queries matched:** 5
gamma-tubulin [Drosophila melanogaster]

gi|17136618 **Mass:** 53306 **Score:** 244 **Queries matched:** 5
gamma-Tubulin at 23C CG3157-PA [Drosophila melanogaster]

---

3. gi|158739 **Mass:** 50119 **Score:** 222 **Queries matched:** 5 **emPAI:** 0.29

beta-1 tubulin

☐ Check to include this hit in error tolerant search or archive report

Query	Observed	Mr(expt)	Mr(calc)	ppm	Miss	Score	Expect	Rank	Peptide
1923	527.3098	1052.6051	1052.6019	3.06	0	42	0.00051	1	R.YLTVAAIFR.G
2062	623.3017	1244.5889	1244.5860	2.37	0	51	7.5e-005	1	R.ISEQFTAMFR.R + Oxidation (M)

	Query	Observed	Mr(expt)	Mr(calc)	ppm	Miss	Score	Expect	Rank	Peptide
☑	2275	809.4142	1616.8138	1616.8080	3.61	0	64	6.8e-006	1	R.AVLVDLEPGTMDSVR.S + Oxidation (M)
☑	2329	854.4365	1706.8584	1706.8549	2.06	0	24	0.069	1	R.ALTVPELTQQMFDAK.N + Oxidation (M)
☑	2425	662.6392	1984.8957	1984.8904	2.66	1	41	0.00078	1	R.MSMKEVDEQMLNIQNK.N + 3 Oxidation (M

Proteins matching the same set of peptides:

gi|24655737 **Mass:** 50115 **Score:** 222 **Queries matched:** 5
beta-Tubulin at 56D CG9277-PB, isoform B [Drosophila melanogaster]

gi|24655741 **Mass:** 51264 **Score:** 222 **Queries matched:** 5
beta-Tubulin at 56D CG9277-PA, isoform A [Drosophila melanogaster]

---

4. gi|20129399 **Mass:** 56983 **Score:** 126 **Queries matched:** 2 **emPAI:** 0.12
Aldehyde dehydrogenase CG3752-PA [Drosophila melanogaster]

☐ Check to include this hit in error tolerant search or archive report

	Query	Observed	Mr(expt)	Mr(calc)	ppm	Miss	Score	Expect	Rank	Peptide
☑	2250	778.3931	1554.7717	1554.7678	2.52	0	93	1e-008	1	R.ANNSEYGLAAAVFTK.D
☑	2357	597.2946	1788.8619	1788.8570	2.74	1	33	0.0064	1	R.TFVEDKIYDEFVER.S

Proteins matching the same set of peptides:

gi|68051705 **Mass:** 61886 **Score:** 126 **Queries matched:** 2
GH22814p [Drosophila melanogaster]

gi|125986975 **Mass:** 56964 **Score:** 126 **Queries matched:** 2
GA17661-PA [Drosophila pseudoobscura]

---

5. gi|6942155 **Mass:** 60188 **Score:** 42 **Queries matched:** 1 **emPAI:** 0.05
putative cytoplasmic aminopeptidase [Drosophila melanogaster]

☐ Check to include this hit in error tolerant search or archive report

	Query	Observed	Mr(expt)	Mr(calc)	ppm	Miss	Score	Expect	Rank	Peptide
☑	1942	543.8117	1085.6089	1085.6081	0.79	0	42	0.00074	1	K.GAQETIALVGK.G

Proteins matching the same set of peptides:

gi|21357381 **Mass:** 60132 **Score:** 42 **Queries matched:** 1

granny smith CG7340-PB, isoform B [Drosophila melanogaster]
gi|24646410 **Mass:** 71134 **Score:** 42 **Queries matched:** 1
granny smith CG7340-PA, isoform A [Drosophila melanogaster]
gi|24646412 **Mass:** 62705 **Score:** 42 **Queries matched:** 1
granny smith CG7340-PC, isoform C [Drosophila melanogaster]
gi|25009916 **Mass:** 71132 **Score:** 42 **Queries matched:** 1
AT31511p [Drosophila melanogaster]
gi|125774513 **Mass:** 56357 **Score:** 42 **Queries matched:** 1
GA20276-PA [Drosophila pseudoobscura]

---

6. gi|1923274 **Mass:** 262206 **Score:** 32 **Queries matched:** 1 **emPAI:** 0.01
Tpr homolog [Drosophila melanogaster]

☐ Check to include this hit in error tolerant search or archive report

	Query	Observed	Mr(expt)	Mr(calc)	ppm	Miss	Score	Expect	Rank	Peptide
☑	1422	718.3640	717.3567	717.3544	3.19	0	32	0.012	1	R.EELAEK.V

Proteins matching the same set of peptides:
gi|24652825 **Mass:** 262194 **Score:** 32 **Queries matched:** 1
Megator CG8274-PA [Drosophila melanogaster]

---

7. gi|17944482 **Mass:** 56834 **Score:** 31 **Queries matched:** 2 **emPAI:** 0.06
RH04336p [Drosophila melanogaster]

☐ Check to include this hit in error tolerant search or archive report

	Query	Observed	Mr(expt)	Mr(calc)	ppm	Miss	Score	Expect	Rank	Peptide
☑	1996	574.3413	1146.6680	1146.6648	2.76	0	(26)	0.0095	1	R.SFAQVEILLK.H
☑	1997	574.3420	1146.6695	1146.6648	4.04	0	31	0.0026	1	R.SFAQVEILLK.H

Proteins matching the same set of peptides:
gi|24659664 **Mass:** 56825 **Score:** 31 **Queries matched:** 2
CG9953-PA [Drosophila melanogaster]

---

8. gi|17945899 **Score:** 31 **Queries matched:** 1

RE40293p [Drosophila melanogaster]

☐ Check to include this hit in error tolerant search or archive report

	Query	Observed	Mr(expt)	Mr(calc)	ppm	Miss	Score	Expect	Rank	Peptide
☑	1214	637.3203	636.3131	636.3119	1.85	0	31	0.0065	1	K.DDFIK.T

Proteins matching the same set of peptides:

gi\|24650758 **Score:** 31 **Queries matched:** 1
gi\|24650760 **Score:** 31 **Queries matched:** 1
gi\|24667151 **Score:** 31 **Queries matched:** 1
gi\|24667155 **Score:** 31 **Queries matched:** 1
gi\|125980730 **Score:** 31 **Queries matched:** 1

---

9. gi\|3426089 **Mass:** 177601 **Score:** 29 **Queries matched:** 5 **emPAI:** 0.02

polyprotein [Drosophila melanogaster]

☐ Check to include this hit in error tolerant search or archive report

	Query	Observed	Mr(expt)	Mr(calc)	ppm	Miss	Score	Expect	Rank	Peptide
☑	1327	672.4047	671.3974	671.3966	1.13	0	(25)	0.049	1	K.TVSLPR.L
☑	1328	672.4049	671.3976	671.3966	1.42	0	29	0.019	1	K.TVSLPR.L
☑	1329	672.4049	671.3976	671.3966	1.51	0	(29)	0.019	1	K.TVSLPR.L
☑	1331	672.4051	671.3978	671.3966	1.78	0	(22)	0.09	1	K.TVSLPR.L
☑	1333	672.4058	671.3985	671.3966	2.77	0	(24)	0.056	1	K.TVSLPR.L

Proteins matching the same set of peptides:

gi\|21214752 **Mass:** 206214 **Score:** 29 **Queries matched:** 5
gag-pol polyprotein precursor; hypothetical protein [Drosophila melanogaster]
gi\|25009978 **Mass:** 83774 **Score:** 29 **Queries matched:** 5
GH06606p [Drosophila melanogaster]

---

10. gi\|304728 **Score:** 28 **Queries matched:** 2

transcription factor

☐ Check to include this hit in error tolerant search or archive report

	Query	Observed	Mr(expt)	Mr(calc)	ppm	Miss	Score	Expect	Rank	Peptide
☑	569	475.2880	474.2807	474.2802	1.12	0	(25)	0.33	1	K.SGIAK.Q
☑	570	475.2881	474.2808	474.2802	1.27	0	28	0.15	1	K.SGIAK.Q

Proteins matching the same set of peptides:

gi|17737765 **Score:** 28 **Queries matched:** 2
gi|28572995 **Score:** 28 **Queries matched:** 2
gi|41616160 **Score:** 28 **Queries matched:** 2
gi|62472391 **Score:** 28 **Queries matched:** 2
gi|92109928 **Score:** 28 **Queries matched:** 2

---

11. gi|19920576 **Mass:** 29241 **Score:** 26 **Queries matched:** 2 **emPAI:** 0.11
CG3557-PA [Drosophila melanogaster]

☐ Check to include this hit in error tolerant search or archive report

	Query	Observed	Mr(expt)	Mr(calc)	ppm	Miss	Score	Expect	Rank	Peptide
☑	1991	572.3413	1142.6680	1142.6713	-2.85	1	(21)	0.094	1	K.WLWTRLIR.G
☑	1992	572.3413	1142.6681	1142.6713	-2.75	1	26	0.033	1	K.WLWTRLIR.G

---

12. gi|21429138 **Mass:** 24766 **Score:** 26 **Queries matched:** 1 **emPAI:** 0.14
RE42508p [Drosophila melanogaster]

☐ Check to include this hit in error tolerant search or archive report

	Query	Observed	Mr(expt)	Mr(calc)	ppm	Miss	Score	Expect	Rank	Peptide
☑	1945	1091.5658	1090.5585	1090.5553	2.96	2	26	0.036	1	R.KMSLEDGRR.A

Proteins matching the same set of peptides:

gi|116007254 **Mass:** 21260 **Score:** 26 **Queries matched:** 1
Rab21 CG17515-PA, isoform A [Drosophila melanogaster]

---

13. gi|9799628 **Score:** 25 **Queries matched:** 2
Nut2 [Drosophila melanogaster]

☐ Check to include this hit in error tolerant search or archive report

	Query	Observed	Mr(expt)	Mr(calc)	ppm	Miss	Score	Expect	Rank	Peptide
☑	1363	687.4418	686.4345	686.4326	2.69	1	(25)	0.072	1	K.IEGLKK.F
☑	1364	687.4433	686.4360	686.4326	4.95	1	25	0.064	1	K.IEGLKK.F

Proteins matching the same set of peptides:
gi|24665095 **Score:** 25 **Queries matched:** 2

---

14. gi|45550970 **Mass:** 154737 **Score:** 25 **Queries matched:** 2 **emPAI:** 0.02
CG5792-PB, isoform B [Drosophila melanogaster]
☐ Check to include this hit in error tolerant search or archive report

	Query	Observed	Mr(expt)	Mr(calc)	ppm	Miss	Score	Expect	Rank	Peptide
☑	1717	432.7330	863.4514	863.4501	1.49	1	(24)	0.062	1	K.LEKFGDR.N
☑	1718	432.7330	863.4515	863.4501	1.56	1	25	0.049	1	K.LEKFGDR.N

Proteins matching the same set of peptides:
gi|90855623 **Mass:** 130026 **Score:** 25 **Queries matched:** 2
IP16022p [Drosophila melanogaster]

---

15. gi|24644950 **Mass:** 36839 **Score:** 25 **Queries matched:** 1
CG2767-PA [Drosophila melanogaster]
☐ Check to include this hit in error tolerant search or archive report

	Query	Observed	Mr(expt)	Mr(calc)	ppm	Miss	Score	Expect	Rank	Peptide
☑	1914	523.2861	1044.5576	1044.5564	1.19	0	25	0.062	1	K.LSSLDQNIR.I

Peptide matches not assigned to protein hits: (no details means no match)

	Query	Observed	Mr(expt)	Mr(calc)	ppm	Miss	Score	Expect	Rank	Peptide
☑	1292	660.3946	659.3874	659.3854	3.05	0	43	0.00085	1	AEISLK
☑	1291	660.3943	659.3870	659.3854	2.55	0	38	0.0028	1	AEISLK
☑	1556	394.7483	787.4820	787.4803	2.11	1	28	0.017	1	LSALEKK
	1669	424.2139	846.4133	846.4157	-2.79	0	24	0.023	1	DPDLMLK + Oxidation (M)

Mascot Search Results

**User**	:	**Julie Kadrmas; gel band: #63**
**Email**	:	**110223FT_JulieKadrmas_63**
**Search title**	:	**NCBI Drosophila DB trypsin specific search: Oxidation (MHW)**
**MS data file**	:	**C:\Documents and Settings\cnelson\Desktop\Merge Files_2011\February_2011\merge_110223FT_JulieKad**
**Database**	:	**NCBInr (5335292 sequences; 1845185051 residues)**
**Taxonomy**	:	**Drosophila (fruit flies) (50248 sequences)**
**Timestamp**	:	**24 Feb 2011 at 19:14:32 GMT**
**Protein hits**	:	**gi|17137738** Rpt1 CG1341-PA [Drosophila melanogaster]
		**gi|20129399** Aldehyde dehydrogenase CG3752-PA [Drosophila melanogaster]
		**gi|24643390** CG32528-PA [Drosophila melanogaster]
		**gi|386043** tubulin alpha-2 chain {internal fragment} [Drosophila melanogaster, CME W2 wing imag
		**gi|1699220** D-ERp60=protein disulphide isomerase isoform/multifunctional endoplasmic reticulum l
		**gi|7946** unnamed protein product [Drosophila melanogaster]
		**gi|45552677** CG33456-PA [Drosophila melanogaster]
		**gi|3426089** polyprotein [Drosophila melanogaster]
		**gi|17946230** RE57556p [Drosophila melanogaster]
		**gi|24644950** CG2767-PA [Drosophila melanogaster]
		**gi|24648138** CG11779-PA, isoform A [Drosophila melanogaster]

Probability Based Mowse Score

Ions score is -10*Log(P), where P is the probability that the observed match is a random event.
Individual ions scores > 23 indicate identity or extensive homology (p<0.05).
Protein scores are derived from ions scores as a non-probabilistic basis for ranking protein hits.

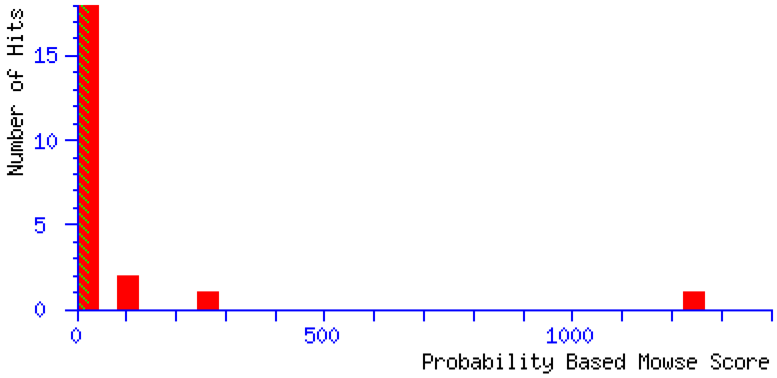

Peptide Summary Report

Format As [Peptide Summary] Help

Significance threshold p< [0.05] Max. number of hits [AUTO]

Standard scoring (•) MudPIT scoring ( ) Ions score or expect cut-off [20] Show sub-sets [0]

Show pop-ups ( ) Suppress pop-ups (•) Sort unassigned [Decreasing Score] Require bold red [x]

Select All | Select None | Search Selected | [ ] **Error tolerant** | Archive Report

1. gi|17137738 **Mass:** 48511 **Score:** 1244 **Queries matched:** 34 **emPAI:** 6.68

Rpt1 CG1341-PA [Drosophila melanogaster]

[ ] Check to include this hit in error tolerant search or archive report

	Query	Observed	Mr(expt)	Mr(calc)	ppm	Miss	Score	Expect	Rank	Peptide
[x]	1621	646.3420	645.3347	645.3333	2.13	0	22	0.13	1	K.EQIEK.L
[x]	2122	456.2190	910.4235	910.4218	1.83	0	30	0.0052	1	R.ELFEMAR.S + Oxidation (M)
[x]	2123	456.2192	910.4238	910.4218	2.17	0	(20)	0.053	1	R.ELFEMAR.S + Oxidation (M)
[x]	2124	456.2193	910.4241	910.4218	2.50	0	(23)	0.026	1	R.ELFEMAR.S + Oxidation (M)
[x]	2158	481.2464	960.4783	960.4764	1.99	0	51	0.00017	1	K.SIEEDIQK.A
[x]	2181	486.7908	971.5670	971.5651	1.91	0	52	0.00016	1	R.VIGSELVQK.Y
	2209	501.2860	1000.5575	1000.5553	2.23	0	40	0.0017	1	K.QVNELTGIK.E

☑

☑	2255	539.7449	1077.4753	1077.4727	2.37	0	51	3e-005	1	M.PDYLGDDQR.K
☑	2260	544.2685	1086.5225	1086.5193	2.94	0	48	7.5e-005	1	K.IINADSDDPK.Y
☑	2277	557.3190	1112.6234	1112.6230	0.41	0	33	0.0044	1	K.FVNLGIEPPK.G
☑	2280	373.5493	1117.6260	1117.6244	1.48	1	(27)	0.018	1	R.DIRFDLLAR.L
☑	2281	559.8207	1117.6269	1117.6244	2.23	1	40	0.00097	1	R.DIRFDLLAR.L
☑	2291	571.8329	1141.6512	1141.6496	1.39	0	36	0.0055	1	K.GVLLFGPPGTGK.T
☑	2327	402.8639	1205.5698	1205.5677	1.81	1	(49)	0.00013	1	M.PDYLGDDQRK.V
☑	2328	603.7931	1205.5717	1205.5677	3.37	1	65	2.9e-006	1	M.PDYLGDDQRK.V
☑	2343	616.3266	1230.6387	1230.6343	3.56	0	55	4.1e-005	1	K.SLDEGDIELLK.T
☑	2344	616.7998	1231.5850	1231.5833	1.35	0	35	0.0038	1	K.VEFGLPDQDGR.S
☑	2352	630.3472	1258.6798	1258.6768	2.38	1	52	9.2e-005	1	K.SIEEDIQKAVK.Q
☑	2353	420.5673	1258.6800	1258.6768	2.54	1	(28)	0.026	1	K.SIEEDIQKAVK.Q
☑	2382	454.2341	1359.6806	1359.6783	1.68	1	(37)	0.0032	1	R.KVEFGLPDQDGR.S
☑	2383	680.8485	1359.6824	1359.6783	3.03	1	48	0.0002	1	R.KVEFGLPDQDGR.S
☑	2389	690.2971	1378.5797	1378.5750	3.43	0	81	4e-008	1	R.FDDGAGGDNEVQR.T
☑	2407	493.2814	1476.8224	1476.8188	2.47	2	51	4.4e-005	1	K.VATEKDFLEAVKK.V
☑	2412	768.9312	1535.8478	1535.8420	3.77	0	61	6.7e-006	1	K.QILQNEQPLQVAR.C
☑	2424	553.9866	1658.9381	1658.9355	1.53	1	29	0.0058	1	K.LREVVETPLLHPEK.F
☑	2432	852.9684	1703.9222	1703.9206	0.90	1	26	0.03	1	R.VIGSELVQKYVGEGAR.M
☑	2437	881.4490	1760.8834	1760.8767	3.80	0	(47)	0.00032	1	R.TMLELINQLDGFDPR.G
☑	2442	889.4456	1776.8766	1776.8716	2.81	0	80	1.7e-007	1	R.TMLELINQLDGFDPR.G + Oxidation (M)
☑	2451	606.6608	1816.9607	1816.9570	1.98	1	(35)	0.0039	1	K.IINADSDDPKYIINVK.Q
☑	2452	909.4882	1816.9618	1816.9570	2.60	1	84	5.1e-008	1	K.IINADSDDPKYIINVK.Q
☑	2481	1040.5005	2078.9865	2078.9830	1.65	0	(80)	1.8e-007	1	K.FVVDLADSVAPTDIEEGMR.V + Oxidation
☑	2482	694.0036	2078.9891	2078.9830	2.89	0	96	4.3e-009	1	K.FVVDLADSVAPTDIEEGMR.V + Oxidation
☑	2496	746.4299	2236.2680	2236.2620	2.67	1	44	0.00014	1	K.FVNLGIEPPKGVLLFGPPGTGK.T
☑	2529	869.4323	2605.2750	2605.2694	2.15	1	36	0.005	1	K.FVVDLADSVAPTDIEEGMRVGVDR.N + Oxida

---

2. gi|20129399 **Mass:** 56983 **Score:** 251 **Queries matched:** 5 **emPAI:** 0.32
Aldehyde dehydrogenase CG3752-PA [Drosophila melanogaster]
☐ Check to include this hit in error tolerant search or archive report

	Query	Observed	Mr(expt)	Mr(calc)	ppm	Miss	Score	Expect	Rank	Peptide
☑	2258	541.2808	1080.5470	1080.5452	1.73	0	52	0.0001	1	K.VAFTGSTDVGK.L
☑	2402	717.3935	1432.7724	1432.7674	3.46	1	50	0.00017	1	K.DLDKANYIVGGLR.A
☑	2415	778.3935	1554.7725	1554.7678	3.02	0	86	4.5e-008	1	R.ANNSEYGLAAAVFTK.D
☑	2446	597.2944	1788.8613	1788.8570	2.38	1	(29)	0.017	1	R.TFVEDKIYDEFVER.S
☑	2447	895.4390	1788.8634	1788.8570	3.58	1	63	6.2e-006	1	R.TFVEDKIYDEFVER.S

Proteins matching the same set of peptides:

gi|68051705 **Mass:** 61886 **Score:** 251 **Queries matched:** 5
GH22814p [Drosophila melanogaster]

gi|125986975 **Mass:** 56964 **Score:** 251 **Queries matched:** 5
GA17661-PA [Drosophila pseudoobscura]

---

3. gi|24643390 **Mass:** 41837 **Score:** 65 **Queries matched:** 1 **emPAI:** 0.08
CG32528-PA [Drosophila melanogaster]

☐ Check to include this hit in error tolerant search or archive report

	Query	Observed	Mr(expt)	Mr(calc)	ppm	Miss	Score	Expect	Rank	Peptide
☑	2422	805.4469	1608.8793	1608.8723	4.36	0	65	2.6e-006	1	R.AVIDPQSISDPQVIK.L

---

4. gi|386043 **Mass:** 1687 **Score:** 63 **Queries matched:** 1 **emPAI:** 2.57
tubulin alpha-2 chain {internal fragment} [Drosophila melanogaster, CME W2 wing imaginal disc cell line, Pep

☐ Check to include this hit in error tolerant search or archive report

	Query	Observed	Mr(expt)	Mr(calc)	ppm	Miss	Score	Expect	Rank	Peptide
☑	2429	844.4505	1686.8864	1686.8829	2.08	0	63	9e-006	1	-.AVFVDLEPTVVDEVR.-

Proteins matching the same set of peptides:

gi|17136564 **Mass:** 49876 **Score:** 63 **Queries matched:** 1
alpha-Tubulin at 84B CG1913-PA [Drosophila melanogaster]

gi|17737821 **Mass:** 49859 **Score:** 63 **Queries matched:** 1
alpha-Tubulin at 84D CG2512-PA, isoform A [Drosophila melanogaster]

gi|17865841 **Mass:** 49935 **Score:** 63 **Queries matched:** 1
alpha-Tubulin at 85E CG9476-PA [Drosophila melanogaster]
gi|21064813 **Mass:** 50034 **Score:** 63 **Queries matched:** 1
RH71862p [Drosophila melanogaster]
gi|38047815 **Mass:** 28524 **Score:** 63 **Queries matched:** 1
similar to Drosophila melanogaster alphaTub84B [Drosophila yakuba]

---

5. gi|1699220 **Mass:** 55338 **Score:** 36 **Queries matched:** 1 **emPAI:** 0.06
D-ERp60=protein disulphide isomerase isoform/multifunctional endoplasmic reticulum luminal polypeptide [Dros

☐ Check to include this hit in error tolerant search or archive report

	Query	Observed	Mr(expt)	Mr(calc)	ppm	Miss	Score	Expect	Rank	Peptide
☑	2279	557.7929	1113.5713	1113.5706	0.63	0	36	0.0035	1	K.YSVSGYPTLK.I

Proteins matching the same set of peptides:
gi|45551086 **Mass:** 55336 **Score:** 36 **Queries matched:** 1
ERp60 CG8983-PA, isoform A [Drosophila melanogaster]
gi|66804043 **Mass:** 55334 **Score:** 36 **Queries matched:** 1
Erp60 [Drosophila simulans]

---

6. gi|7946 **Mass:** 46534 **Score:** 32 **Queries matched:** 1 **emPAI:** 0.07
unnamed protein product [Drosophila melanogaster]

☐ Check to include this hit in error tolerant search or archive report

	Query	Observed	Mr(expt)	Mr(calc)	ppm	Miss	Score	Expect	Rank	Peptide
☑	2233	522.7832	1043.5518	1043.5499	1.85	0	32	0.0086	1	R.IEEEIGAGVK.F

Proteins matching the same set of peptides:
gi|17137654 **Mass:** 46633 **Score:** 32 **Queries matched:** 1
Enolase CG17654-PA, isoform A [Drosophila melanogaster]
gi|21392248 **Mass:** 54304 **Score:** 32 **Queries matched:** 1
SD23356p [Drosophila melanogaster]
gi|24580914 **Mass:** 54276 **Score:** 32 **Queries matched:** 1
Enolase CG17654-PB, isoform B [Drosophila melanogaster]

---

7. gi|45552677 **Mass:** 74415 **Score:** 32 **Queries matched:** 1 **emPAI:** 0.04
CG33456-PA [Drosophila melanogaster]

☐ Check to include this hit in error tolerant search or archive report

	Query	Observed	Mr(expt)	Mr(calc)	ppm	Miss	Score	Expect	Rank	Peptide
☑	2244	530.7865	1059.5585	1059.5560	2.33	0	32	0.013	1	R.TLIDQESVR.D

---

8. gi|3426089 **Mass:** 177601 **Score:** 32 **Queries matched:** 3 **emPAI:** 0.02
polyprotein [Drosophila melanogaster]

☐ Check to include this hit in error tolerant search or archive report

	Query	Observed	Mr(expt)	Mr(calc)	ppm	Miss	Score	Expect	Rank	Peptide
☑	1715	672.4047	671.3974	671.3966	1.13	0	(31)	0.01	1	K.TVSLPR.L
☑	1716	672.4049	671.3976	671.3966	1.42	0	(27)	0.028	1	K.TVSLPR.L
☑	1718	672.4054	671.3981	671.3966	2.24	0	32	0.01	1	K.TVSLPR.L

Proteins matching the same set of peptides:
gi|21214752 **Mass:** 206214 **Score:** 32 **Queries matched:** 3
gag-pol polyprotein precursor; hypothetical protein [Drosophila melanogaster]
gi|25009978 **Mass:** 83774 **Score:** 32 **Queries matched:** 3
GH06606p [Drosophila melanogaster]

---

9. gi|17946230 **Mass:** 43594 **Score:** 25 **Queries matched:** 1 **emPAI:** 0.08
RE57556p [Drosophila melanogaster]

☐ Check to include this hit in error tolerant search or archive report

	Query	Observed	Mr(expt)	Mr(calc)	ppm	Miss	Score	Expect	Rank	Peptide
☑	2076	866.4293	865.4220	865.4181	4.50	0	25	0.033	1	K.EYADLQK.R

Proteins matching the same set of peptides:
gi|24584126 **Mass:** 43654 **Score:** 25 **Queries matched:** 1
CG9267-PA [Drosophila melanogaster]

10. gi|24644950 **Mass:** 36839 **Score:** 25 **Queries matched:** 2
CG2767-PA [Drosophila melanogaster]
☐ Check to include this hit in error tolerant search or archive report

	Query	Observed	Mr(expt)	Mr(calc)	ppm	Miss	Score	Expect	Rank	Peptide
☑	2235	523.2860	1044.5575	1044.5564	1.08	0	25	0.064	1	K.LSSLDQNIR.I
☑	2236	523.2864	1044.5583	1044.5564	1.83	0	(25)	0.065	1	K.LSSLDQNIR.I

11. gi|24648138 **Mass:** 52842 **Score:** 25 **Queries matched:** 1 **emPAI:** 0.06
CG11779-PA, isoform A [Drosophila melanogaster]
☐ Check to include this hit in error tolerant search or archive report

	Query	Observed	Mr(expt)	Mr(calc)	ppm	Miss	Score	Expect	Rank	Peptide
☑	1961	787.4594	786.4522	786.4487	4.40	0	25	0.045	1	R.GDLEILK.D

Proteins matching the same set of peptides:
gi|24648140 **Mass:** 48895 **Score:** 25 **Queries matched:** 1
CG11779-PB, isoform B [Drosophila melanogaster]
gi|25009742 **Mass:** 52856 **Score:** 25 **Queries matched:** 1
AT09326p [Drosophila melanogaster]
gi|63093891 **Mass:** 52816 **Score:** 25 **Queries matched:** 1
hypothetical protein [Drosophila parabipectinata]
gi|63093892 **Mass:** 48814 **Score:** 25 **Queries matched:** 1
hypothetical protein [Drosophila parabipectinata]
gi|78706782 **Mass:** 49345 **Score:** 25 **Queries matched:** 1
CG11779-PC, isoform C [Drosophila melanogaster]
gi|125773847 **Mass:** 51199 **Score:** 25 **Queries matched:** 1
GA11194-PA [Drosophila pseudoobscura]

Peptide matches not assigned to protein hits: (no details means no match)

	Query	Observed	Mr(expt)	Mr(calc)	ppm	Miss	Score	Expect	Rank	Peptide
☑	2351	630.3420	1258.6695	1258.6710	-1.22	0	22	0.1	1	SLPFSPSHFLK
	2042	423.7203	845.4261	845.4243	2.14	0	21	0.13	1	ALAEAESR

MATRIX SCIENCE Mascot Search Results

```
User            : Julie Kadrmas; gel band: #64
Email           : 110224FT_JulieKadrmas_64
Search title    : NCBI Drosophila DB trypsin specific search: Oxidation (MHW)
MS data file    : C:\Documents and Settings\cnelson\Desktop\Merge Files_2011\February_2011\merge_110224FT_JulieKad
Database        : NCBInr  (5335292 sequences; 1845185051 residues)
Taxonomy        : Drosophila (fruit flies) (50248 sequences)
Timestamp       : 24 Feb 2011 at 19:39:26 GMT
Protein hits    : gi|24643390  CG32528-PA [Drosophila melanogaster]
                  gi|62862344  CG10837-PB.3 [Drosophila melanogaster]
                  gi|3426089   polyprotein [Drosophila melanogaster]
                  gi|66771585  IP12293p [Drosophila melanogaster]
                  gi|63146399  rhino [Drosophila simulans]
                  gi|125811483 GA21181-PA [Drosophila pseudoobscura]
```

Probability Based Mowse Score

Ions score is -10*Log(P), where P is the probability that the observed match is a random event.
Individual ions scores > 24 indicate identity or extensive homology ($p<0.05$).
Protein scores are derived from ions scores as a non-probabilistic basis for ranking protein hits.

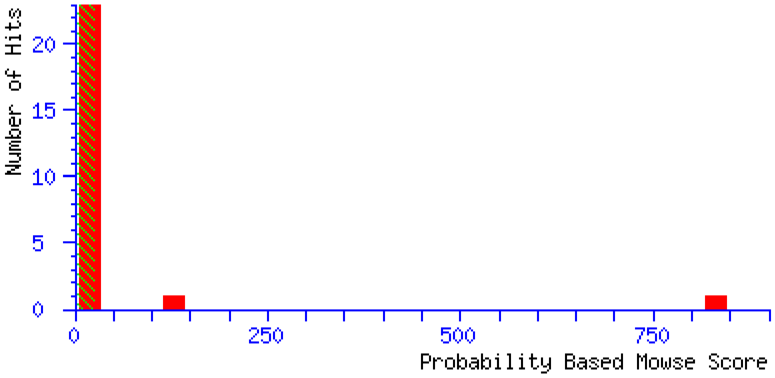

Peptide Summary Report

Format As: Peptide Summary — Help

Significance threshold p< 0.05 — Max. number of hits AUTO

Standard scoring (selected) — MudPIT scoring — Ions score or expect cut-off 20 — Show sub-sets 0

Show pop-ups — Suppress pop-ups (selected) — Sort unassigned Decreasing Score — Require bold red (checked)

Select All — Select None — Search Selected — Error tolerant — Archive Report

1. gi|24643390 **Mass:** 41837 **Score:** 832 **Queries matched:** 21 **emPAI:** 3.57
CG32528-PA [Drosophila melanogaster]

Check to include this hit in error tolerant search or archive report

Query	Observed	Mr(expt)	Mr(calc)	ppm	Miss	Score	Expect	Rank	Peptide
1589	350.2425	698.4704	698.4690	2.00	0	40	0.00037	1	K.LNIVLK.A
1590	699.4780	698.4708	698.4690	2.45	0	(28)	0.0063	1	K.LNIVLK.A
1842	457.7573	913.5000	913.4981	2.06	0	33	0.0064	1	K.NAGVLNAQK.F
1848	461.2746	920.5346	920.5331	1.66	0	47	0.00019	1	K.SLITFVNK.H
1887	500.7650	999.5155	999.5138	1.76	0	49	9.5e-005	1	K.WSVASVHSK.N
1919	525.3225	1048.6305	1048.6281	2.29	1	56	1.2e-005	1	K.KSLITFVNK.H
1980	417.8921	1250.6545	1250.6520	2.02	0	36	0.0036	1	K.AVNHTLGFHQK.I
1981	626.3352	1250.6558	1250.6520	3.03	0	(36)	0.0045	1	K.AVNHTLGFHQK.I
1985	646.8401	1291.6657	1291.6635	1.70	0	80	1.5e-007	1	R.VLYSLFTMFR.D + Oxidation (M)
1996	456.8869	1367.6390	1367.6357	2.37	2	27	0.022	1	K.KGEKEDSFWDK.F
1999	472.9110	1415.7111	1415.7078	2.28	0	(42)	0.00083	1	K.ARPEDIVNMDLK.S + Oxidation (M)
2000	708.8635	1415.7124	1415.7078	3.20	0	48	0.00024	1	K.ARPEDIVNMDLK.S + Oxidation (M)
2012	779.4503	1556.8861	1556.8814	3.01	0	69	9e-007	1	R.LPENVFVTVVIAEK.N
2018	537.2992	1608.8757	1608.8723	2.10	0	(57)	2.5e-005	1	R.AVIDPQSISDPQVIK.L
2019	805.4462	1608.8778	1608.8723	3.42	0	67	2e-006	1	R.AVIDPQSISDPQVIK.L
2020	805.4467	1608.8789	1608.8723	4.14	0	(64)	3.5e-006	1	R.AVIDPQSISDPQVIK.L
2030	857.4455	1712.8765	1712.8733	1.83	0	82	9.7e-008	1	R.ILVDWINDELAEQR.I
2038	924.4119	1846.8092	1846.8043	2.62	0	98	7.6e-010	1	K.FQEQITSEYDDLGMR.C + Oxidation (M)
2039	616.6106	1846.8099	1846.8043	2.99	0	(65)	1.9e-006	1	K.FQEQITSEYDDLGMR.C + Oxidation (M)
2051	651.3306	1950.9699	1950.9647	2.68	1	66	4.1e-006	1	K.KLDVPEVTQSEQGQHEK.L

	Query	Observed	Mr(expt)	Mr(calc)	ppm	Miss	Score	Expect	Rank	Peptide
☑										
☑	2064	728.3658	2182.0755	2182.0728	1.23	0	34	0.0072	1	R.IIVQHLEEDMYDGQVLHK.L + Oxidation (M

---

2. gi|62862344 **Mass:** 52171 **Score:** 131 **Queries matched:** 3 **emPAI:** 0.20
CG10837-PB.3 [Drosophila melanogaster]

☐ Check to include this hit in error tolerant search or archive report

	Query	Observed	Mr(expt)	Mr(calc)	ppm	Miss	Score	Expect	Rank	Peptide
☑	1845	458.7419	915.4692	915.4661	3.37	0	23	0.055	1	R.ELEIEQR.L
☑	2002	716.8398	1431.6650	1431.6590	4.22	0	83	6.2e-008	1	R.IELSNENDQQSR.Q
☑	2015	528.2625	1581.7657	1581.7634	1.44	2	25	0.047	1	R.TNQDYKESDKINK.C

Proteins matching the same set of peptides:
gi|62862346 **Mass:** 44097 **Score:** 131 **Queries matched:** 3
CG10837-PE.3 [Drosophila melanogaster]
gi|62862348 **Mass:** 44197 **Score:** 131 **Queries matched:** 3
CG10837-PA.3 [Drosophila melanogaster]

---

3. gi|3426089 **Mass:** 177601 **Score:** 31 **Queries matched:** 3 **emPAI:** 0.02
polyprotein [Drosophila melanogaster]

☐ Check to include this hit in error tolerant search or archive report

	Query	Observed	Mr(expt)	Mr(calc)	ppm	Miss	Score	Expect	Rank	Peptide
☑	1530	672.4047	671.3974	671.3966	1.13	0	(27)	0.029	1	K.TVSLPR.L
☑	1531	672.4047	671.3974	671.3966	1.18	0	31	0.01	1	K.TVSLPR.L
☑	1532	672.4049	671.3977	671.3966	1.55	0	(31)	0.01	1	K.TVSLPR.L

Proteins matching the same set of peptides:
gi|21214752 **Mass:** 206214 **Score:** 31 **Queries matched:** 3
gag-pol polyprotein precursor; hypothetical protein [Drosophila melanogaster]
gi|25009978 **Mass:** 83774 **Score:** 31 **Queries matched:** 3
GH06606p [Drosophila melanogaster]

---

4. gi|66771585 **Mass:** 7369 **Score:** 31 **Queries matched:** 1 **emPAI:** 0.48
IP12293p [Drosophila melanogaster]
☐ Check to include this hit in error tolerant search or archive report

	Query	Observed	Mr(expt)	Mr(calc)	ppm	Miss	Score	Expect	Rank	Peptide
☑	1732	401.7252	801.4359	801.4345	1.85	1	31	0.026	1	K.DLGRTLAG.-

5. gi|63146399 **Mass:** 55283 **Score:** 25 **Queries matched:** 1
rhino [Drosophila simulans]
☐ Check to include this hit in error tolerant search or archive report

	Query	Observed	Mr(expt)	Mr(calc)	ppm	Miss	Score	Expect	Rank	Peptide
☑	1773	845.4495	844.4422	844.4403	2.35	1	25	0.053	1	K.SSSPPKSR.G

6. gi|125811483 **Mass:** 144257 **Score:** 25 **Queries matched:** 1 **emPAI:** 0.02
GA21181-PA [Drosophila pseudoobscura]
☐ Check to include this hit in error tolerant search or archive report

	Query	Observed	Mr(expt)	Mr(calc)	ppm	Miss	Score	Expect	Rank	Peptide
☑	1400	628.3429	627.3356	627.3340	2.56	0	25	0.051	1	R.AHGISK.Q + Oxidation (HW)

Peptide matches not assigned to protein hits: (no details means no match)

	Query	Observed	Mr(expt)	Mr(calc)	ppm	Miss	Score	Expect	Rank	Peptide
☑	1852	466.7444	931.4742	931.4770	-3.04	2	24	0.072	1	ACSPRRSR
☑	1856	466.7448	931.4751	931.4770	-2.02	2	23	0.098	1	ACSPRRSR
☑	1921	530.7869	1059.5592	1059.5560	3.03	0	23	0.1	1	TLIDQESVR
☑	1763	416.7486	831.4826	831.4814	1.42	0	21	0.096	1	SLSLSLGR
☑	1778	846.4351	845.4278	845.4243	4.14	0	21	0.15	1	NNLDSVGK
☑	1772	423.2281	844.4417	844.4443	-3.09	0	20	0.16	1	SPALWGSK
☑	1290	586.3934	585.3861	585.3850	1.93	0	20	0.11	1	GIGVIK
☑	1240	573.3362	572.3289	572.3282	1.25	0	20	0.34	1	INAQK
☑	1898	510.7725	1019.5305	1019.5321	-1.64	0	20	0.16	1	AIMVGTVGEK + Oxidation (M)

Mascot Search Results

**User**	:	**Julie Kadrmas; gel band: #66**
**Email**	:	**110224FT_JulieKadrmas_66**
**Search title**	:	**NCBI Drosophila DB trypsin specific search: Oxidation (MHW)**
**MS data file**	:	**C:\Documents and Settings\cnelson\Desktop\Merge Files_2011\February_2011\merge_110224FT_JulieKad**
**Database**	:	**NCBInr (5335292 sequences; 1845185051 residues)**
**Taxonomy**	:	**Drosophila (fruit flies) (50248 sequences)**
**Timestamp**	:	**25 Feb 2011 at 18:43:33 GMT**
**Protein hits**	:	**gi|21357547** CG8863-PA, isoform A [Drosophila melanogaster]
		**gi|158767** ubiquitin
		**gi|157891** myosin heavy chain
		**gi|3426089** polyprotein [Drosophila melanogaster]
		**gi|3915856** 40S ribosomal protein S3a (C3 protein)
		**gi|1150726** transcription factor
		**gi|41619832** TPA: TPA_inf: HDC15749 [Drosophila melanogaster]
		**gi|45552677** CG33456-PA [Drosophila melanogaster]
		**gi|125982242** GA22115-PA [Drosophila pseudoobscura]
		**gi|19922798** CG3085-PA [Drosophila melanogaster]
		**gi|24668309** CG11248-PA, isoform A [Drosophila melanogaster]

Probability Based Mowse Score

Ions score is -10*Log(P), where P is the probability that the observed match is a random event.
Individual ions scores > 23 indicate identity or extensive homology (p<0.05).
Protein scores are derived from ions scores as a non-probabilistic basis for ranking protein hits.

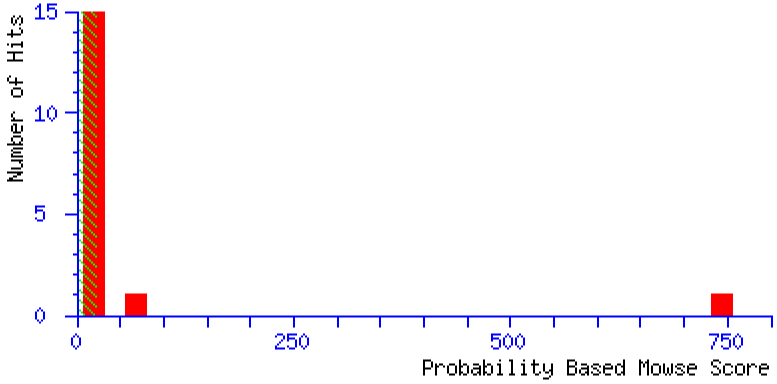

Peptide Summary Report

Format As | Peptide Summary | Help

Significance threshold p< 0.05 | Max. number of hits AUTO

Standard scoring ◉ MudPIT scoring ○ | Ions score or expect cut-off 20 | Show sub-sets 0

Show pop-ups ○ Suppress pop-ups ◉ | Sort unassigned Decreasing Score | Require bold red ☑

Select All | Select None | Search Selected | ☐ **Error tolerant** | Archive Report

1. gi|21357547 **Mass:** 45202 **Score:** 743 **Queries matched:** 24 **emPAI:** 2.82
CG8863-PA, isoform A [Drosophila melanogaster]
☐ Check to include this hit in error tolerant search or archive report

	Query	Observed	Mr(expt)	Mr(calc)	ppm	Miss	Score	Expect	Rank	Peptide
☑	2430	379.2510	756.4875	756.4857	2.27	1	30	0.022	1	R.KLQLQK.N
☑	2785	483.7856	965.5567	965.5546	2.22	0	45	0.00019	1	K.VLEVHIEK.G
☑	2878	365.5578	1093.6516	1093.6495	1.88	1	(31)	0.003	1	R.KVLEVHIEK.G
☑	2879	547.8334	1093.6523	1093.6495	2.50	1	39	0.00056	1	R.KVLEVHIEK.G
☑	2928	587.2765	1172.5384	1172.5363	1.77	0	57	1.2e-005	1	K.FFGAGFGGSGGGR.R
☑	2994	640.3137	1278.6129	1278.6092	2.91	0	62	6.3e-006	1	R.QVYDEGGEAAIK.K
	3034	469.9095	1406.7067	1406.7041	1.83	1	(55)	4.6e-005	1	R.QVYDEGGEAAIKK.G

☑										
☑	3035	704.3610	1406.7074	1406.7041	2.30	1	63	7.9e-006	1	R.QVYDEGGEAAIKK.G
☑	3041	714.3345	1426.6545	1426.6477	4.75	1	23	0.044	1	K.YHPDKNPNEGEK.F
☑	3092	555.9539	1664.8397	1664.8369	1.69	1	(30)	0.023	1	K.AISQAYEVLSDADKR.Q
☑	3093	833.4276	1664.8406	1664.8369	2.21	1	(74)	9.5e-007	1	K.AISQAYEVLSDADKR.Q
☑	3094	833.4284	1664.8422	1664.8369	3.16	1	87	5e-008	1	K.AISQAYEVLSDADKR.Q
☑	3105	851.9160	1701.8174	1701.8111	3.74	2	21	0.12	1	K.YHPDKNPNEGEKFK.A
☑	3106	852.8282	1703.6419	1703.6369	2.93	0	82	6.8e-009	1	R.MAYDEDDGGYQDGPR.V + Oxidation (M)
☑	3138	618.3261	1851.9565	1851.9479	4.67	2	31	0.013	1	K.LALKYHPDKNPNEGEK.F
☑	3140	635.9423	1904.8050	1904.7999	2.63	1	34	0.00083	1	K.GGADSGDFRNPMDFFEK.F + Oxidation (M)
☑	3141	635.9429	1904.8070	1904.7999	3.69	1	(27)	0.0059	1	K.GGADSGDFRNPMDFFEK.F + Oxidation (M)
☑	3167	676.3653	2026.0742	2026.0695	2.30	1	33	0.0047	1	K.TLDDRDLIVSTQPGEVIR.H
☑	3168	678.6398	2032.8977	2032.8949	1.36	2	(20)	0.066	1	K.KGGADSGDFRNPMDFFEK.F + Oxidation (M
☑	3169	678.6418	2032.9037	2032.8949	4.34	2	32	0.0051	1	K.KGGADSGDFRNPMDFFEK.F + Oxidation (M
☑	3226	797.3996	2389.1770	2389.1696	3.08	1	49	0.00026	1	R.GKDVVHQMSVQLEELYNGATR.K + Oxidation
☑	3234	885.1814	2652.5224	2652.5142	3.06	0	28	0.0028	1	K.GTLIIQFEVIFPEVINPSVVPTLK.Q
☑	3235	890.4575	2668.3505	2668.3490	0.57	2	(24)	0.077	1	K.TLDDRDLIVSTQPGEVIRHEMTK.C + Oxidati
☑	3236	890.4596	2668.3569	2668.3490	2.93	2	32	0.01	1	K.TLDDRDLIVSTQPGEVIRHEMTK.C + Oxidati

---

2. gi|158767 **Mass:** 8540 **Score:** 45 **Queries matched:** 1 **emPAI:** 0.41
ubiquitin

☐ Check to include this hit in error tolerant search or archive report

	Query	Observed	Mr(expt)	Mr(calc)	ppm	Miss	Score	Expect	Rank	Peptide
☑	3126	894.4700	1786.9255	1786.9200	3.04	0	45	0.00089	1	K.TITLEVEPSDTIENVK.A

Proteins matching the same set of peptides:

gi|158769 **Mass:** 8428 **Score:** 45 **Queries matched:** 1
ubiquitin
gi|158771 **Mass:** 8543 **Score:** 45 **Queries matched:** 1
ubiquitin
gi|17136570 **Mass:** 14720 **Score:** 45 **Queries matched:** 1
Ribosomal protein L40 CG2960-PA [Drosophila melanogaster]

gi|17136574 **Mass:** 17929 **Score:** 45 **Queries matched:** 1
Ribosomal protein S27A CG5271-PA [Drosophila melanogaster]
gi|51701919 **Mass:** 8560 **Score:** 45 **Queries matched:** 1
Ubiquitin
gi|158763 **Score:** 43 **Queries matched:** 1
gi|158765 **Score:** 43 **Queries matched:** 1
gi|225319 **Score:** 43 **Queries matched:** 1
gi|225320 **Score:** 43 **Queries matched:** 1
gi|24640094 **Mass:** 34314 **Score:** 43 **Queries matched:** 1
CG11700-PA [Drosophila melanogaster]
gi|158753 **Mass:** 25955 **Score:** 42 **Queries matched:** 1
ubiquitin
gi|21428336 **Mass:** 34369 **Score:** 42 **Queries matched:** 1
GH17513p [Drosophila melanogaster]
gi|24640086 **Mass:** 59993 **Score:** 40 **Queries matched:** 1
CG32744-PA [Drosophila melanogaster]
gi|24657014 **Mass:** 85746 **Score:** 39 **Queries matched:** 1
Ubiquitin-63E CG11624-PA, isoform A [Drosophila melanogaster]
gi|21429768 **Mass:** 119913 **Score:** 39 **Queries matched:** 1
AT20865p [Drosophila melanogaster]

---

3. gi|157891 **Mass:** 224288 **Score:** 31 **Queries matched:** 1 **emPAI:** 0.01
myosin heavy chain

☐ Check to include this hit in error tolerant search or archive report

	Query	Observed	Mr(expt)	Mr(calc)	ppm	Miss	Score	Expect	Rank	Peptide
☑	2530	**819.3864**	**818.3791**	**818.3770**	**2.59**	**0**	**31**	**0.0089**	**1**	**R.LADEESR.E**

Proteins matching the same set of peptides:
gi|157892 **Mass:** 224488 **Score:** 31 **Queries matched:** 1
myosin heavy chain
gi|2546936 **Mass:** 135522 **Score:** 31 **Queries matched:** 1
muscle myosin heavy chain [Drosophila melanogaster]
gi|2546937 **Mass:** 135517 **Score:** 31 **Queries matched:** 1
muscle myosin heavy chain [Drosophila melanogaster]

gi|2546938 **Mass:** 138464 **Score:** 31 **Queries matched:** 1
muscle myosin heavy chain [Drosophila melanogaster]
gi|2546939 **Mass:** 138459 **Score:** 31 **Queries matched:** 1
muscle myosin heavy chain [Drosophila melanogaster]
gi|7579901 **Mass:** 44936 **Score:** 31 **Queries matched:** 1
myosin heavy chain; MHC [Drosophila melanogaster]
gi|24584692 **Mass:** 224278 **Score:** 31 **Queries matched:** 1
Myosin heavy chain CG17927-PC, isoform C [Drosophila melanogaster]
gi|24584694 **Mass:** 224395 **Score:** 31 **Queries matched:** 1
Myosin heavy chain CG17927-PG, isoform G [Drosophila melanogaster]
gi|24584696 **Mass:** 224496 **Score:** 31 **Queries matched:** 1
Myosin heavy chain CG17927-PE, isoform E [Drosophila melanogaster]
gi|24584698 **Mass:** 224269 **Score:** 31 **Queries matched:** 1
Myosin heavy chain CG17927-PJ, isoform J [Drosophila melanogaster]
gi|24584700 **Mass:** 224391 **Score:** 31 **Queries matched:** 1
Myosin heavy chain CG17927-PF, isoform F [Drosophila melanogaster]
gi|24584702 **Mass:** 224355 **Score:** 31 **Queries matched:** 1
Myosin heavy chain CG17927-PD, isoform D [Drosophila melanogaster]
gi|24584704 **Mass:** 224472 **Score:** 31 **Queries matched:** 1
Myosin heavy chain CG17927-PA, isoform A [Drosophila melanogaster]
gi|24584706 **Mass:** 224456 **Score:** 31 **Queries matched:** 1
Myosin heavy chain CG17927-PI, isoform I [Drosophila melanogaster]
gi|24584710 **Mass:** 224272 **Score:** 31 **Queries matched:** 1
Myosin heavy chain CG17927-PB, isoform B [Drosophila melanogaster]
gi|24584712 **Mass:** 221271 **Score:** 31 **Queries matched:** 1
Myosin heavy chain CG17927-PK, isoform K [Drosophila melanogaster]
gi|24584714 **Mass:** 221339 **Score:** 31 **Queries matched:** 1
Myosin heavy chain CG17927-PL, isoform L [Drosophila melanogaster]
gi|24584716 **Mass:** 221330 **Score:** 31 **Queries matched:** 1
Myosin heavy chain CG17927-PM, isoform M [Drosophila melanogaster]
gi|28574239 **Mass:** 224573 **Score:** 31 **Queries matched:** 1
Myosin heavy chain CG17927-PH, isoform H [Drosophila melanogaster]

---

4. gi|3426089 **Mass:** 177601 **Score:** 29 **Queries matched:** 4 **emPAI:** 0.02
polyprotein [Drosophila melanogaster]
☐ Check to include this hit in error tolerant search or archive report

	Query	Observed	Mr(expt)	Mr(calc)	ppm	Miss	Score	Expect	Rank	Peptide
☑	2177	672.4046	671.3974	671.3966	1.09	0	(27)	0.028	1	K.TVSLPR.L
☑	2178	672.4051	671.3978	671.3966	1.82	0	29	0.02	1	K.TVSLPR.L
☑	2180	672.4052	671.3980	671.3966	2.00	0	(23)	0.079	1	K.TVSLPR.L
☑	2181	672.4055	671.3982	671.3966	2.37	0	(28)	0.022	1	K.TVSLPR.L

Proteins matching the same set of peptides:
gi|21214752 **Mass:** 206214 **Score:** 29 **Queries matched:** 4
gag-pol polyprotein precursor; hypothetical protein [Drosophila melanogaster]
gi|25009978 **Mass:** 83774 **Score:** 29 **Queries matched:** 4
GH06606p [Drosophila melanogaster]

---

5. gi|3915856 **Mass:** 30337 **Score:** 28 **Queries matched:** 1 **emPAI:** 0.11
40S ribosomal protein S3a (C3 protein)
☐ Check to include this hit in error tolerant search or archive report

	Query	Observed	Mr(expt)	Mr(calc)	ppm	Miss	Score	Expect	Rank	Peptide
☑	2510	405.2244	808.4342	808.4330	1.48	0	28	0.014	1	R.IASDYLK.G

Proteins matching the same set of peptides:
gi|17864162 **Mass:** 30321 **Score:** 28 **Queries matched:** 1
Ribosomal protein S3A CG2168-PA, isoform A [Drosophila melanogaster]
gi|17945018 **Mass:** 25085 **Score:** 28 **Queries matched:** 1
RE04220p [Drosophila melanogaster]
gi|38047857 **Mass:** 30307 **Score:** 28 **Queries matched:** 1
similar to Drosophila melanogaster RpS3A [Drosophila yakuba]
gi|38048393 **Mass:** 25024 **Score:** 28 **Queries matched:** 1
similar to Drosophila melanogaster RpS3A [Drosophila yakuba]
gi|126002490 **Mass:** 30307 **Score:** 28 **Queries matched:** 1
GA15280-PA [Drosophila pseudoobscura]

---

6. gi|1150726 **Mass:** 112844 **Score:** 26 **Queries matched:** 1
transcription factor

☐ Check to include this hit in error tolerant search or archive report

	Query	Observed	Mr(expt)	Mr(calc)	ppm	Miss	Score	Expect	Rank	Peptide
☑	2556	416.2510	830.4874	830.4862	1.55	0	26	0.067	1	R.SLVVANTK.T

Proteins matching the same set of peptides:

gi|24664166 **Mass:** 112804 **Score:** 26 **Queries matched:** 1
stonewall CG3836-PA [Drosophila melanogaster]
gi|125979313 **Mass:** 107544 **Score:** 26 **Queries matched:** 1
GA17720-PA [Drosophila pseudoobscura]

---

7. gi|41619832 **Mass:** 27811 **Score:** 24 **Queries matched:** 1 **emPAI:** 0.12
TPA: TPA_inf: HDC15749 [Drosophila melanogaster]

☐ Check to include this hit in error tolerant search or archive report

	Query	Observed	Mr(expt)	Mr(calc)	ppm	Miss	Score	Expect	Rank	Peptide
☑	2870	541.7511	1081.4877	1081.4896	-1.75	1	24	0.034	1	K.MEMEKVQR.V + 2 Oxidation (M)

---

8. gi|45552677 **Mass:** 74415 **Score:** 24 **Queries matched:** 1 **emPAI:** 0.04
CG33456-PA [Drosophila melanogaster]

☐ Check to include this hit in error tolerant search or archive report

	Query	Observed	Mr(expt)	Mr(calc)	ppm	Miss	Score	Expect	Rank	Peptide
☑	2858	530.7866	1059.5587	1059.5560	2.51	0	24	0.084	1	R.TLIDQESVR.D

---

9. gi|125982242 **Mass:** 50218 **Score:** 24 **Queries matched:** 1
GA22115-PA [Drosophila pseudoobscura]

☐ Check to include this hit in error tolerant search or archive report

	Query	Observed	Mr(expt)	Mr(calc)	ppm	Miss	Score	Expect	Rank	Peptide
☑	2501	401.7252	801.4359	801.4344	1.77	1	24	0.16	1	R.EREQIK.R

---

10. gi|19922798 **Mass:** 50665 **Score:** 23 **Queries matched:** 1
CG3085-PA [Drosophila melanogaster]
☐ Check to include this hit in error tolerant search or archive report

	Query	Observed	Mr(expt)	Mr(calc)	ppm	Miss	Score	Expect	Rank	Peptide
☑	2380	731.4070	730.3998	730.3973	3.34	0	23	0.22	1	R.ISELNR.W

Proteins matching the same set of peptides:
gi|27374258 **Mass:** 50627 **Score:** 23 **Queries matched:** 1
CG3085-PA [Drosophila erecta]
gi|125807457 **Mass:** 50658 **Score:** 23 **Queries matched:** 1
GA15915-PA [Drosophila pseudoobscura]

---

11. gi|24668309 **Mass:** 96012 **Score:** 23 **Queries matched:** 1 **emPAI:** 0.03
CG11248-PA, isoform A [Drosophila melanogaster]
☐ Check to include this hit in error tolerant search or archive report

	Query	Observed	Mr(expt)	Mr(calc)	ppm	Miss	Score	Expect	Rank	Peptide
☑	2584	846.4335	845.4262	845.4243	2.29	0	23	0.089	1	K.ALAEAESR.I

Peptide matches not assigned to protein hits: (no details means no match)

	Query	Observed	Mr(expt)	Mr(calc)	ppm	Miss	Score	Expect	Rank	Peptide
☑	1656	559.3458	558.3385	558.3377	1.42	0	22	0.17	1	IAEVK
☑	2741	466.7448	931.4751	931.4770	-1.99	2	21	0.15	1	ACSPRRSR
☑	2738	466.7435	931.4724	931.4770	-4.94	2	21	0.14	1	ACSPRRSR
☑	1990	625.3202	624.3130	624.3119	1.76	0	21	0.099	1	SYLDK
☑	2490	794.3867	793.3794	793.3817	-2.88	1	20	0.092	1	SSSEKEK
☑	2511	405.2246	808.4346	808.4330	1.93	0	20	0.068	1	IASDYLK
☑	2509	807.4024	806.3951	806.3956	-0.63	0	20	0.15	1	MSSPTLR + Oxidation (M)
☑	2557	831.4952	830.4879	830.4862	2.10	0	20	0.25	1	SLVVANTK
☑	1657	559.3466	558.3393	558.3377	2.96	0	20	0.3	1	IAEVK
☑	2789	487.2707	972.5268	972.5240	2.86	0	20	0.17	1	LLTDELNR
☑	2011	628.3781	627.3708	627.3704	0.68	0	19	0.15	1	GSVLPR

Mascot Search Results

**User**	:	**Julie Kadrmas; gel band: #92**
**Email**	:	**110224FT_JulieKadrmas_92**
**Search title**	:	**NCBI Drosophila DB trypsin specific search: Oxidation (MHW)**
**MS data file**	:	**C:\Documents and Settings\cnelson\Desktop\Merge Files_2011\February_2011\merge_110224FT_JulieKad**
**Database**	:	**NCBInr (5335292 sequences; 1845185051 residues)**
**Taxonomy**	:	**Drosophila (fruit flies) (50248 sequences)**
**Timestamp**	:	**25 Feb 2011 at 18:51:51 GMT**
**Protein hits**	:	gi|17737405 Proteasome 35kD subunit CG4904-PA [Drosophila melanogaster]
		gi|62862344 CG10837-PB.3 [Drosophila melanogaster]
		gi|17136226 Ecdysone-inducible gene L3 CG10160-PA [Drosophila melanogaster]
		gi|82466274 kl-2 protein [Drosophila bipectinata]
		gi|3426089 polyprotein [Drosophila melanogaster]
		gi|24653473 CG30484-PA [Drosophila melanogaster]
		gi|8630 unnamed protein product [Drosophila melanogaster]
		gi|125985389 GA19998-PA [Drosophila pseudoobscura]
		gi|21358649 CG3508-PA, isoform A [Drosophila melanogaster]
		gi|16768410 GM04011p [Drosophila melanogaster]

Probability Based Mowse Score

Ions score is -10*Log(P), where P is the probability that the observed match is a random event.
Individual ions scores > 24 indicate identity or extensive homology (p<0.05).
Protein scores are derived from ions scores as a non-probabilistic basis for ranking protein hits.

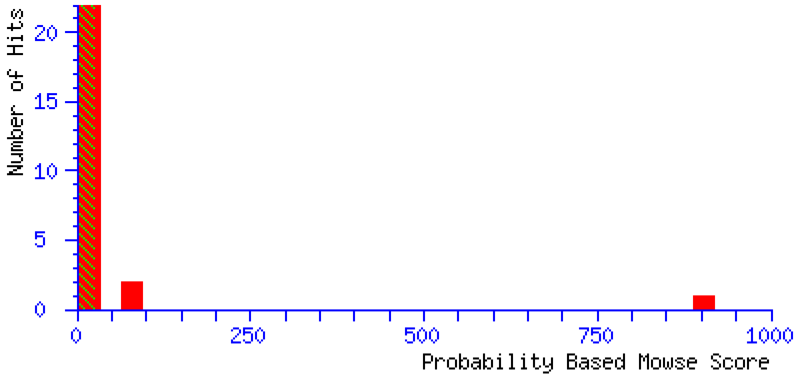

Peptide Summary Report

Format As | Peptide Summary | Help

Significance threshold p< 0.05 | Max. number of hits AUTO

Standard scoring ◉ MudPIT scoring ○ | Ions score or expect cut-off 20 | Show sub-sets 0

Show pop-ups ○ Suppress pop-ups ◉ | Sort unassigned Decreasing Score | Require bold red ☑

Select All | Select None | Search Selected | ☐ **Error tolerant** | Archive Report

1. gi|17737405 **Mass:** 31040 **Score:** 905 **Queries matched:** 33 **emPAI:** 10.46
Proteasome 35kD subunit CG4904-PA [Drosophila melanogaster]
☐ Check to include this hit in error tolerant search or archive report

	Query	Observed	Mr(expt)	Mr(calc)	ppm	Miss	Score	Expect	Rank	Peptide
☑	1356	638.4000	637.3927	637.3911	2.44	0	29	0.0077	1	K.HVAIAK.E
☑	1376	645.3580	644.3507	644.3493	2.13	0	24	0.14	1	K.DEIIR.H
☑	1407	653.3519	652.3447	652.3432	2.29	0	24	0.019	1	R.TYLEK.N
☑	1762	436.7645	871.5145	871.5127	2.04	0	41	0.0015	1	R.LITNLGNK.M
☑	1763	872.5224	871.5151	871.5127	2.79	0	(37)	0.004	1	R.LITNLGNK.M
☑	1764	872.5232	871.5160	871.5127	3.76	0	(33)	0.0096	1	R.LITNLGNK.M
	1967	561.8131	1121.6116	1121.6080	3.18	1	30	0.0066	1	R.TYLEKNLNK.F

☑										
☑	1980	581.8100	1161.6054	1161.6030	2.12	0	39	0.002	1	K.DQPFTILSNK.D
☑	2041	441.5707	1321.6903	1321.6878	1.89	1	(47)	0.00032	1	K.FLDSSKDEIIR.H
☑	2042	661.8534	1321.6923	1321.6878	3.43	1	66	2.8e-006	1	K.FLDSSKDEIIR.H
☑	2043	663.3113	1324.6080	1324.6048	2.43	0	56	2.5e-005	1	K.HSYDTTYPVSR.L
☑	2048	671.8659	1341.7172	1341.7140	2.39	0	53	7.4e-005	1	R.AILGTLPTDEQGK.D
☑	2052	677.3563	1352.6981	1352.6936	3.33	0	84	5.7e-008	1	R.DPEVLVATEQRP.-
☑	2065	478.5754	1432.7043	1432.7020	1.59	0	(29)	0.019	1	R.LHQVEYAMEAVK.L + Oxidation (M)
☑	2066	478.5758	1432.7057	1432.7020	2.55	0	(45)	0.0004	1	R.LHQVEYAMEAVK.L + Oxidation (M)
☑	2067	717.3605	1432.7064	1432.7020	3.05	0	45	0.00051	1	R.LHQVEYAMEAVK.L + Oxidation (M)
☑	2096	782.4060	1562.7974	1562.7940	2.16	1	(23)	0.073	1	K.DQPFTILSNKDSAK.H
☑	2097	521.9399	1562.7979	1562.7940	2.48	1	35	0.0055	1	K.DQPFTILSNKDSAK.H
☑	2117	578.9785	1733.9136	1733.9094	2.43	1	(48)	0.00031	1	R.LITNLGNKMQTTTQR.Y + Oxidation (M)
☑	2118	867.9645	1733.9145	1733.9094	2.92	1	72	1.3e-006	1	R.LITNLGNKMQTTTQR.Y + Oxidation (M)
☑	2119	867.9646	1733.9146	1733.9094	2.98	1	(51)	0.00017	1	R.LITNLGNKMQTTTQR.Y + Oxidation (M)
☑	2122	876.4050	1750.7954	1750.7911	2.45	0	(61)	9.2e-006	1	R.NQYDSDVTVWSPQGR.L
☑	2123	584.6061	1750.7966	1750.7911	3.14	0	(40)	0.001	1	R.NQYDSDVTVWSPQGR.L
☑	2124	876.4057	1750.7968	1750.7911	3.25	0	67	1.9e-006	1	R.NQYDSDVTVWSPQGR.L
☑	2133	884.4024	1766.7903	1766.7860	2.44	0	(47)	0.00015	1	R.NQYDSDVTVWSPQGR.L + Oxidation (HW)
☑	2134	884.4025	1766.7905	1766.7860	2.54	0	(64)	2.9e-006	1	R.NQYDSDVTVWSPQGR.L + Oxidation (HW)
☑	2143	597.9941	1790.9606	1790.9526	4.45	2	48	0.00027	1	K.NLNKFLDSSKDEIIR.H
☑	2167	687.7181	2060.1324	2060.1266	2.82	0	(31)	0.0063	1	K.IIPIDDHLGISIAGLTADAR.V
☑	2168	1031.0739	2060.1332	2060.1266	3.22	0	96	2.1e-009	1	K.IIPIDDHLGISIAGLTADAR.V
☑	2169	687.7188	2060.1344	2060.1266	3.80	0	(32)	0.0046	1	K.IIPIDDHLGISIAGLTADAR.V
☑	2170	1031.0756	2060.1366	2060.1266	4.88	0	(77)	1.5e-007	1	K.IIPIDDHLGISIAGLTADAR.V
☑	2176	700.3624	2098.0655	2098.0596	2.80	1	29	0.022	1	R.YDRRPYGVGLLVAGYDER.G
☑	2183	730.4163	2188.2270	2188.2215	2.47	1	69	5.6e-007	1	R.KIIPIDDHLGISIAGLTADAR.V

---

2. gi|62862344 **Mass:** 52171 **Score:** 62 **Queries matched:** 2 **emPAI:** 0.13
CG10837-PB.3 [Drosophila melanogaster]

☐ Check to include this hit in error tolerant search or archive report

Query Observed Mr(expt) Mr(calc) ppm Miss Score Expect Rank Peptide

	Query	Observed	Mr(expt)	Mr(calc)	ppm	Miss	Score	Expect	Rank	Peptide
☑	1830	458.7409	915.4672	915.4661	1.17	0	26	0.033	1	R.ELEIEQR.L
☑	2111	851.4325	1700.8505	1700.8441	3.75	1	36	0.0046	1	R.IRIELSNENDQQSR.Q

Proteins matching the same set of peptides:

gi|62862346 **Mass:** 44097 **Score:** 62 **Queries matched:** 2
CG10837-PE.3 [Drosophila melanogaster]

gi|62862348 **Mass:** 44197 **Score:** 62 **Queries matched:** 2
CG10837-PA.3 [Drosophila melanogaster]

---

3. gi|17136226 **Mass:** 35515 **Score:** 53 **Queries matched:** 1 **emPAI:** 0.09
Ecdysone-inducible gene L3 CG10160-PA [Drosophila melanogaster]

☐ Check to include this hit in error tolerant search or archive report

	Query	Observed	Mr(expt)	Mr(calc)	ppm	Miss	Score	Expect	Rank	Peptide
☑	2091	763.9261	1525.8376	1525.8352	1.62	0	53	5.8e-005	1	K.QILTPTEVEQLQK.S

Proteins matching the same set of peptides:

gi|78214230 **Mass:** 35524 **Score:** 53 **Queries matched:** 1
RE54418p [Drosophila melanogaster]

---

4. gi|82466274 **Mass:** 19078 **Score:** 35 **Queries matched:** 1 **emPAI:** 0.18
kl-2 protein [Drosophila bipectinata]

☐ Check to include this hit in error tolerant search or archive report

	Query	Observed	Mr(expt)	Mr(calc)	ppm	Miss	Score	Expect	Rank	Peptide
☑	1907	515.3014	1028.5882	1028.5866	1.54	0	35	0.0047	1	K.DELVLTIAR.N

Proteins matching the same set of peptides:

gi|82466286 **Mass:** 19064 **Score:** 35 **Queries matched:** 1
kl-2 protein [Drosophila pseudoananassae nigrens]

---

5. gi|3426089 **Mass:** 177601 **Score:** 31 **Queries matched:** 3 **emPAI:** 0.02
polyprotein [Drosophila melanogaster]

☐ Check to include this hit in error tolerant search or archive report

	Query	Observed	Mr(expt)	Mr(calc)	ppm	Miss	Score	Expect	Rank	Peptide
☑	1453	672.4046	671.3974	671.3966	1.09	0	31	0.01	1	K.TVSLPR.L
☑	1456	672.4051	671.3978	671.3966	1.78	0	(29)	0.019	1	K.TVSLPR.L
☑	1457	672.4051	671.3978	671.3966	1.82	0	(31)	0.011	1	K.TVSLPR.L

Proteins matching the same set of peptides:

gi|21214752 **Mass:** 206214 **Score:** 31 **Queries matched:** 3
gag-pol polyprotein precursor; hypothetical protein [Drosophila melanogaster]
gi|25009978 **Mass:** 83774 **Score:** 31 **Queries matched:** 3
GH06606p [Drosophila melanogaster]

---

6. gi|24653473 **Mass:** 101560 **Score:** 30 **Queries matched:** 1 **emPAI:** 0.03
CG30484-PA [Drosophila melanogaster]

☐ Check to include this hit in error tolerant search or archive report

	Query	Observed	Mr(expt)	Mr(calc)	ppm	Miss	Score	Expect	Rank	Peptide
☑	1812	453.7388	905.4631	905.4640	-1.06	1	30	0.019	1	K.MLEGKANK.I + Oxidation (M)

---

7. gi|8630 **Mass:** 42958 **Score:** 27 **Queries matched:** 1 **emPAI:** 0.08
unnamed protein product [Drosophila melanogaster]

☐ Check to include this hit in error tolerant search or archive report

	Query	Observed	Mr(expt)	Mr(calc)	ppm	Miss	Score	Expect	Rank	Peptide
☑	1522	697.3347	696.3274	696.3265	1.41	0	27	0.016	1	-.MAANYK.S

Proteins matching the same set of peptides:

gi|17136490 **Mass:** 42928 **Score:** 27 **Queries matched:** 1
snail CG3956-PA [Drosophila melanogaster]
gi|125984502 **Mass:** 44640 **Score:** 27 **Queries matched:** 1
GA17803-PA [Drosophila pseudoobscura]

---

8. gi|125985389 **Mass:** 238718 **Score:** 26 **Queries matched:** 1 **emPAI:** 0.01
GA19998-PA [Drosophila pseudoobscura]
☐ Check to include this hit in error tolerant search or archive report

	Query	Observed	Mr(expt)	Mr(calc)	ppm	Miss	Score	Expect	Rank	Peptide
☑	1895	501.7645	1001.5144	1001.5141	0.24	1	28	0.046	1	K.AEEEQRLK.A

---

9. gi|21358649 **Mass:** 38035 **Score:** 25 **Queries matched:** 2 **emPAI:** 0.09
CG3508-PA, isoform A [Drosophila melanogaster]
☐ Check to include this hit in error tolerant search or archive report

	Query	Observed	Mr(expt)	Mr(calc)	ppm	Miss	Score	Expect	Rank	Peptide
☑	1446	664.3349	663.3276	663.3261	2.24	0	25	0.046	1	-.MAEAVK.N + Oxidation (M)
☑	1447	664.3359	663.3286	663.3261	3.71	0	(21)	0.11	1	-.MAEAVK.N + Oxidation (M)

Proteins matching the same set of peptides:
gi|24646884 **Mass:** 39187 **Score:** 25 **Queries matched:** 2
CG3508-PB, isoform B [Drosophila melanogaster]
gi|25012395 **Mass:** 39158 **Score:** 25 **Queries matched:** 2
RE11603p [Drosophila melanogaster]
gi|125774419 **Mass:** 34857 **Score:** 25 **Queries matched:** 2
GA17490-PA [Drosophila pseudoobscura]

---

10. gi|16768410 **Score:** 24 **Queries matched:** 1
GM04011p [Drosophila melanogaster]
☐ Check to include this hit in error tolerant search or archive report

	Query	Observed	Mr(expt)	Mr(calc)	ppm	Miss	Score	Expect	Rank	Peptide
☑	1031	545.3055	544.2982	544.2969	2.41	0	24	0.28	1	R.NLGNK.F

Proteins matching the same set of peptides:
gi|24582135 **Score:** 24 **Queries matched:** 1
gi|45580808 **Score:** 24 **Queries matched:** 1

Peptide matches not assigned to protein hits: (no details means no match)

	Query	Observed	Mr(expt)	Mr(calc)	ppm	Miss	Score	Expect	Rank	Peptide
☑	1987	588.3181	1174.6216	1174.6167	4.22	2	24	0.06	1	RASSQSQQKR
☑	1836	466.7445	931.4745	931.4770	-2.65	2	23	0.076	1	ACSPRRSR
☑	1386	646.3533	645.3460	645.3446	2.25	0	23	0.17	1	NTLNGK
☑	1767	437.7542	873.4939	873.4920	2.26	1	22	0.11	1	DGKINLSK
☑	1919	523.2869	1044.5592	1044.5564	2.72	0	22	0.12	1	LSSLDQNIR
☑	1859	947.5165	946.5093	946.5056	3.82	2	22	0.09	1	SSRAQSRR
☑	1766	874.4898	873.4826	873.4807	2.10	0	21	0.12	1	SLVEAEVK
☑	1846	468.2707	934.5268	934.5236	3.47	1	21	0.11	1	AAYDKVLR
☑	1724	423.2281	844.4417	844.4443	-3.09	0	20	0.15	1	SPALWGSK
☑	1725	423.2283	844.4420	844.4443	-2.72	0	20	0.16	1	SPALWGSK
☑	1860	474.2621	946.5097	946.5058	4.06	0	20	0.14	1	TWIMIQR
☑	888	515.3307	514.3234	514.3227	1.40	0	20	0.49	1	LGAVR
☑	1525	699.4147	698.4075	698.4075	-0.09	0	19	0.19	1	QVSLPR
☑	1755	432.7511	863.4877	863.4899	-2.49	1	19	0.16	1	SMLTKIR + Oxidation (M)
☑	1778	439.7332	877.4519	877.4480	4.46	0	19	0.19	1	LMQWLR + Oxidation (HW); Oxidation (M
☑	1408	653.3521	652.3448	652.3432	2.50	0	19	0.059	1	TYLEK
☑	1715	421.2614	840.5083	840.5069	1.71	0	18	0.072	1	IGGPGSLIK
☑	1671	791.4434	790.4361	790.4371	-1.19	1	18	0.47	1	QMLKQK + Oxidation (M)
☑	1698	827.3995	826.3923	826.3895	3.39	0	18	0.16	1	MEAAYVK + Oxidation (M)
☑	1026	543.3514	542.3442	542.3428	2.54	0	18	0.11	1	SPLVK
☑	1918	523.2862	1044.5579	1044.5564	1.48	0	18	0.32	1	LSSLDQNIR
☑	886	515.3307	514.3234	514.3227	1.34	0	18	0.81	1	LGAVR
☑	889	515.3308	514.3235	514.3227	1.52	0	17	0.83	1	LGAVR
☑	891	515.3309	514.3236	514.3227	1.73	0	17	0.83	1	LGAVR
☑	887	515.3307	514.3234	514.3227	1.40	0	17	0.83	1	LGAVR
☑	1455	672.4049	671.3976	671.3966	1.51	0	17	0.26	1	TVSLPR
☑	890	515.3308	514.3235	514.3227	1.56	0	17	0.84	1	LGAVR
☑	1729	423.7202	845.4259	845.4243	1.99	0	17	0.36	1	ALAEAESR
☑	1428	663.3853	662.3780	662.3752	4.35	0	17	0.42	1	GQAFLK
☑	1427	663.3851	662.3778	662.3752	3.99	0	17	0.44	1	GQAFLK

Mascot Search Results

```
User          : Julie Kadrmas; gel band: #111
Email         : 110224FT_JulieKadrmas_111
Search title  : NCBI Drosophila DB trypsin specific search: Oxidation (MHW)
MS data file  : C:\Documents and Settings\cnelson\Desktop\Merge Files_2011\February_2011\merge_110224FT_JulieKad
Database      : NCBInr  (5335292 sequences; 1845185051 residues)
Taxonomy      : Drosophila (fruit flies) (50248 sequences)
Timestamp     : 25 Feb 2011 at 18:54:28 GMT
Protein hits  : gi|21428580 LD43891p [Drosophila melanogaster]
                gi|24644197 CG11999-PA [Drosophila melanogaster]
                gi|2582508  20S proteasome alpha7 subunit [Drosophila melanogaster]
                gi|24584030 CG16972-PA [Drosophila melanogaster]
                gi|3426089  polyprotein [Drosophila melanogaster]
                gi|17862878 SD01389p [Drosophila melanogaster]
                gi|8380     unnamed protein product [Drosophila melanogaster]
                gi|24644950 CG2767-PA [Drosophila melanogaster]
```

Probability Based Mowse Score

Ions score is -10*Log(P), where P is the probability that the observed match is a random event.
Individual ions scores > 24 indicate identity or extensive homology (p<0.05).
Protein scores are derived from ions scores as a non-probabilistic basis for ranking protein hits.

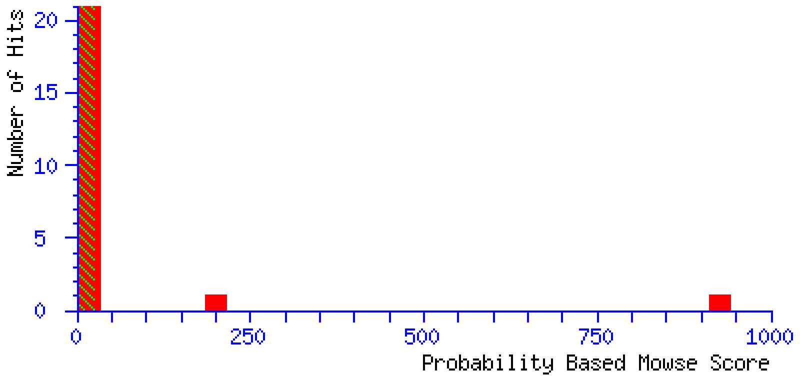

Peptide Summary Report

Format As | Peptide Summary | Help

Significance threshold p< 0.05 | Max. number of hits AUTO

Standard scoring ◉ MudPIT scoring ○ | Ions score or expect cut-off 20 | Show sub-sets 0

Show pop-ups ○ Suppress pop-ups ◉ | Sort unassigned Decreasing Score | Require bold red ☑

Select All | Select None | Search Selected | ☐ **Error tolerant** | Archive Report

1. gi|21428580 **Mass:** 30698 **Score:** 925 **Queries matched:** 34 **emPAI:** 12.03
LD43891p [Drosophila melanogaster]
☐ Check to include this hit in error tolerant search or archive report

	Query	Observed	Mr(expt)	Mr(calc)	ppm	Miss	Score	Expect	Rank	Peptide
☑	1534	673.3901	672.3828	672.3806	3.23	0	25	0.056	1	K.EVGQLK.N
☑	1604	702.3796	701.3724	701.3708	2.25	0	(24)	0.1	1	K.VLDEAR.E
☑	1606	702.3803	701.3730	701.3708	3.12	0	32	0.015	1	K.VLDEAR.E
☑	1733	787.4331	786.4258	786.4236	2.88	0	26	0.04	1	R.EVGDLVR.L
☑	1763	406.7358	811.4570	811.4552	2.24	0	33	0.0046	1	R.LTLSHNK.I
☑	1794	415.7409	829.4673	829.4657	1.87	1	(37)	0.0044	1	K.KVLDEAR.E
☑	1795	415.7412	829.4679	829.4657	2.64	1	42	0.0013	1	K.KVLDEAR.E
☑	1886	463.7932	925.5719	925.5709	1.08	0	46	0.00013	1	K.NLQILGLR.D
☑	1887	463.7938	925.5731	925.5709	2.40	0	(36)	0.0011	1	K.NLQILGLR.D
☑	1888	464.7831	927.5516	927.5501	1.56	0	(39)	0.0015	1	R.ILNVSINR.L
☑	1889	464.7831	927.5516	927.5501	1.59	0	(59)	1.6e-005	1	R.ILNVSINR.L
☑	1891	464.7832	927.5519	927.5501	1.88	0	60	1.4e-005	1	R.ILNVSINR.L
☑	1892	464.7837	927.5529	927.5501	2.97	0	(59)	1.5e-005	1	R.ILNVSINR.L
☑	1944	497.2732	992.5319	992.5291	2.83	1	47	0.00029	1	R.SGPPPPKADK.S
☑	1952	512.2713	1022.5281	1022.5257	2.35	0	27	0.04	1	R.ELHIQNNR.L
☑	1969	542.7868	1083.5591	1083.5560	2.86	0	63	4.8e-006	1	R.DNDLLELPR.E
☑	1993	599.3775	1196.7405	1196.7353	4.30	1	49	4.9e-005	1	K.LRILNVSINR.L
☑	2009	431.5785	1291.7137	1291.7109	2.21	1	37	0.002	1	R.LRELHIQNNR.L

☑	2010	646.8644	1291.7142	1291.7109	2.58	1	(33)	0.0045	1	R.LRELHIQNNR.L
☑	2014	650.8500	1299.6855	1299.6823	2.51	1	39	0.0022	1	K.TETYKIIYNR.H
☑	2022	461.2256	1380.6548	1380.6521	1.98	0	(33)	0.0047	1	R.ETHNPELDLADK.G
☑	2023	691.3359	1380.6573	1380.6521	3.74	0	69	1e-006	1	R.ETHNPELDLADK.G
☑	2037	545.6759	1634.0059	1633.9991	4.14	1	47	2.2e-005	1	R.ILNVSINRLINLPR.G
☑	2038	829.4126	1656.8107	1656.8035	4.32	0	63	7.4e-006	1	R.ALYLGDNDFEYIPK.E
☑	2060	618.3314	1851.9723	1851.9690	1.79	1	36	0.0036	1	R.DNDLLELPREVGDLVR.L
☑	2061	946.0439	1890.0733	1890.0826	-4.92	0	(38)	0.00053	1	R.LQVLPPEIAQLDLLSNK.S
☑	2062	631.0356	1890.0850	1890.0826	1.29	0	(53)	1.8e-005	1	R.LQVLPPEIAQLDLLSNK.S
☑	2063	946.0505	1890.0865	1890.0826	2.05	0	54	1.5e-005	1	R.LQVLPPEIAQLDLLSNK.S
☑	2064	946.0511	1890.0876	1890.0826	2.64	0	(47)	6.5e-005	1	R.LQVLPPEIAQLDLLSNK.S
☑	2072	996.5666	1991.1186	1991.1163	1.12	1	49	7.8e-005	1	K.NLQILGLRDNDLLELPR.E
☑	2073	664.7149	1991.1228	1991.1163	3.24	1	(41)	0.00035	1	K.NLQILGLRDNDLLELPR.E
☑	2080	689.0135	2064.0186	2064.0123	3.05	1	34	0.0078	1	K.VLDEARETHNPELDLADK.G
☑	2083	1064.5235	2127.0325	2127.0306	0.88	0	(45)	0.00047	1	K.GLSSFEELPGLFNMSNITR.L + Oxidation
☑	2084	710.0181	2127.0326	2127.0306	0.90	0	49	0.00022	1	K.GLSSFEELPGLFNMSNITR.L + Oxidation

Proteins matching the same set of peptides:

gi|45550188 **Mass:** 31830 **Score:** 925 **Queries matched:** 34

Ras suppressor-1 CG9031-PA [Drosophila melanogaster]

---

2. gi|24644197 **Mass:** 23619 **Score:** 188 **Queries matched:** 4 **emPAI:** 0.70

CG11999-PA [Drosophila melanogaster]

☐ Check to include this hit in error tolerant search or archive report

	Query	Observed	Mr(expt)	Mr(calc)	ppm	Miss	Score	Expect	Rank	Peptide
☑	1975	549.7837	1097.5529	1097.5505	2.15	0	45	0.00034	1	K.LLNSDYAFR.L
☑	2017	681.3785	1360.7424	1360.7391	2.44	0	57	2.5e-005	1	R.WTTAEGLFIVPK.E
☑	2020	688.2942	1374.5738	1374.5688	3.63	0	27	0.0047	1	K.SSTHDEYAHSEL.-
☑	2049	580.2861	1737.8364	1737.8291	4.22	1	61	1.3e-005	1	R.LRHIDTGMYLGMSGR.S + 2 Oxidation (M)

---

3. gi|2582508 **Mass:** 27444 **Score:** 43 **Queries matched:** 1 **emPAI:** 0.12

20S proteasome alpha7 subunit [Drosophila melanogaster]

☐ Check to include this hit in error tolerant search or archive report

	Query	Observed	Mr(expt)	Mr(calc)	ppm	Miss	Score	Expect	Rank	Peptide
☑	1966	535.7814	1069.5482	1069.5444	3.54	0	43	0.00056	1	R.VFQIDYASK.V

Proteins matching the same set of peptides:

gi\|24652204 **Mass:** 27658 **Score:** 43 **Queries matched:** 1
Proteasome alpha7 subunit CG1519-PA, isoform A [Drosophila melanogaster]

gi\|28573936 **Mass:** 25656 **Score:** 43 **Queries matched:** 1
Proteasome alpha7 subunit CG1519-PB, isoform B [Drosophila melanogaster]

gi\|125811303 **Mass:** 53546 **Score:** 43 **Queries matched:** 1
GA13558-PA [Drosophila pseudoobscura]

---

4. gi\|24584030 **Score:** 38 **Queries matched:** 1
CG16972-PA [Drosophila melanogaster]

☐ Check to include this hit in error tolerant search or archive report

	Query	Observed	Mr(expt)	Mr(calc)	ppm	Miss	Score	Expect	Rank	Peptide
☑	1138	558.3253	557.3180	557.3173	1.35	0	38	0.0025	1	K.KPADK.S

---

5. gi\|3426089 **Mass:** 177601 **Score:** 29 **Queries matched:** 5 **emPAI:** 0.02
polyprotein [Drosophila melanogaster]

☐ Check to include this hit in error tolerant search or archive report

	Query	Observed	Mr(expt)	Mr(calc)	ppm	Miss	Score	Expect	Rank	Peptide
☑	1527	672.4048	671.3975	671.3966	1.27	0	29	0.019	1	K.TVSLPR.L
☑	1528	672.4049	671.3976	671.3966	1.46	0	(24)	0.051	1	K.TVSLPR.L
☑	1529	672.4054	671.3981	671.3966	2.24	0	(29)	0.019	1	K.TVSLPR.L
☑	1530	672.4055	671.3982	671.3966	2.37	0	(28)	0.023	1	K.TVSLPR.L
☑	1532	672.4060	671.3988	671.3966	3.19	0	(28)	0.021	1	K.TVSLPR.L

Proteins matching the same set of peptides:

gi\|21214752 **Mass:** 206214 **Score:** 29 **Queries matched:** 5

gag-pol polyprotein precursor; hypothetical protein [Drosophila melanogaster]
gi|25009978 **Mass:** 83774 **Score:** 29 **Queries matched:** 5
GH06606p [Drosophila melanogaster]

---

6. gi|17862878 **Mass:** 232888 **Score:** 28 **Queries matched:** 1 **emPAI:** 0.01
SD01389p [Drosophila melanogaster]

☐ Check to include this hit in error tolerant search or archive report

	Query	Observed	Mr(expt)	Mr(calc)	ppm	Miss	Score	Expect	Rank	Peptide
☑	1537	674.3734	673.3662	673.3646	2.26	0	28	0.027	1	K.IDLADK.E

Proteins matching the same set of peptides:
gi|19527661 **Mass:** 141204 **Score:** 28 **Queries matched:** 1
SD03956p [Drosophila melanogaster]
gi|24650843 **Mass:** 232920 **Score:** 28 **Queries matched:** 1
CG10011-PA [Drosophila melanogaster]
gi|125773229 **Mass:** 229479 **Score:** 28 **Queries matched:** 1
GA10007-PA [Drosophila pseudoobscura]

---

7. gi|8380 **Mass:** 29436 **Score:** 25 **Queries matched:** 1 **emPAI:** 0.11
unnamed protein product [Drosophila melanogaster]

☐ Check to include this hit in error tolerant search or archive report

	Query	Observed	Mr(expt)	Mr(calc)	ppm	Miss	Score	Expect	Rank	Peptide
☑	1967	536.8000	1071.5855	1071.5812	4.03	0	25	0.031	1	K.LLDSAIPSEK.I

Proteins matching the same set of peptides:
gi|17136420 **Mass:** 29393 **Score:** 25 **Queries matched:** 1
Proteasome 29kD subunit CG9327-PA [Drosophila melanogaster]
gi|125811518 **Mass:** 29205 **Score:** 25 **Queries matched:** 1
GA21704-PA [Drosophila pseudoobscura]

---

8. gi|24644950 **Mass:** 36839 **Score:** 25 **Queries matched:** 2
CG2767-PA [Drosophila melanogaster]

☐ Check to include this hit in error tolerant search or archive report

	Query	Observed	Mr(expt)	Mr(calc)	ppm	Miss	Score	Expect	Rank	Peptide
☑	1957	523.2861	1044.5577	1044.5564	1.30	0	25	0.063	1	K.LSSLDQNIR.I
☑	1959	523.2873	1044.5601	1044.5564	3.58	0	(22)	0.14	1	K.LSSLDQNIR.I

Peptide matches not assigned to protein hits: (no details means no match)

	Query	Observed	Mr(expt)	Mr(calc)	ppm	Miss	Score	Expect	Rank	Peptide
☑	781	499.2993	498.2920	498.2914	1.20	0	23	0.12	1	GGIPR
☑	782	499.2996	498.2923	498.2914	1.72	0	23	0.13	1	GGIPR
☑	1300	603.3106	602.3034	602.3024	1.65	0	22	0.22	1	DLEAR
☑	1330	612.3845	611.3772	611.3755	2.80	0	21	0.1	1	LNIPR
☑	1170	571.3574	570.3501	570.3489	2.11	0	21	0.05	1	VSIPR
☑	1881	920.4873	919.4800	919.4763	4.05	1	21	0.18	1	EKYPVER
☑	1302	603.3113	602.3040	602.3024	2.77	0	21	0.32	1	DLEAR
☑	1809	423.2281	844.4416	844.4443	-3.19	0	20	0.16	1	SPALWGSK
☑	1304	603.3116	602.3043	602.3024	3.16	0	20	0.37	1	DLEAR
☑	1650	725.4692	724.4620	724.4595	3.35	0	20	0.06	1	LINLPR
☑	1732	394.2197	786.4249	786.4236	1.66	0	19	0.19	1	EVGDLVR
☑	1895	466.7426	931.4706	931.4723	-1.80	0	19	0.16	1	INDSLQSR
☑	2110	771.4011	2311.1815	2311.1736	3.44	1	19	0.23	1	ALYLGDNDFEYIPKEVGQLK
☑	1382	628.3793	627.3721	627.3704	2.63	0	19	0.16	1	GSVLPR
☑	1605	702.3798	701.3725	701.3708	2.50	0	19	0.3	1	VLDEAR
☑	1890	928.5590	927.5518	927.5501	1.76	0	19	0.17	1	ILNVSINR
☑	1303	603.3114	602.3041	602.3024	2.92	0	19	0.49	1	DLEAR
☑	1168	571.3568	570.3495	570.3489	0.99	0	19	0.087	1	VSIPR
☑	1489	658.3905	657.3832	657.3810	3.46	0	18	0.44	1	EAAIVR
☑	1535	673.3908	672.3835	672.3806	4.32	0	18	0.27	1	EVGQLK
☑	1566	681.3808	680.3735	680.3718	2.54	0	18	0.062	1	HLQAGR
☑	1989	587.8335	1173.6525	1173.6506	1.62	0	18	0.18	1	YVLPLGTQQR
☑	922	515.3309	514.3237	514.3227	1.81	0	17	0.83	1	LGAVR
☑	920	515.3309	514.3236	514.3227	1.69	0	17	0.84	1	LGAVR
