# Supplementary material for: Interactions by 2D Gel Electrophoresis Overlap (iGEO): a novel high fidelity approach to identify constituents of protein complexes
Source: Proteome Sci. 2013 May 12;11:21. doi: 10.1186/1477-5956-11-21 (PMC3688448; doi:10.1186/1477-5956-11-21)
Supplement: Additional file 8: Method S2 — Detailed procedures of LC-MS/MS. [file 1477-5956-11-21-S8.doc]

**Method S2: Detailed procedures of LC-MS/MS**

# In-gel Digestion

For in-gel digestion, gel bands/spots of interest were excised then de-stained twice in 1 ml volumes of 50% methanol with 50 mM ammonium bicarbonate (NH_4_HCO_3_) at room temperature while being gently vortexed for about 1 hour. The gel slices/spots were re-hydrated in 1 ml of 50 mM NH_4_HCO_3_ for 30 min at room temperature then the gel bands/spots were cut into several pieces. The gel pieces were dehydrated in 1 ml of 100% acetonitrile for 30 min at room temperature with gentle shaking. The acetonitrile was carefully removed away from the gel pieces with a pipette tip prior to proteolytic digestion. 10 to 20 μL of sequence-grade modified trypsin (Promega) (20 ng/μL) in 50 mM NH_4_HCO_3_ was added and adsorbed into the gel pieces and incubated overnight at 37^o^C. The digestion was quenched by the addition of 20 μL ml of 1% formic acid. This solution was allowed to stand and peptides that dissolved in the 1% formic solution were extracted and collected. Further extraction of peptides from the gel material was performed twice by the addition of 50% acetonitrile with 1% formic acid and sonicated at 37^o^C for 20 minutes, and these solutions were also collected and combined. A final complete dehydration of the gel pieces was accomplished by addition of 20 μL of 100% acetonitrile and incubation at 37^o^C for 20 minutes. The supernatant solutions of extracted peptides were combined. Peptides were dried then reconstituted in 100 μL of 5% acetonitrile with 0.1% formic acid for LC/MS/MS analysis.

**Mass Spectrometry (LC/MS/MS)**

Peptides were analyzed using a nano-LC/MS/MS system comprised of a nano-LC pump (2D-ultra, Eksigent) and a LTQ-FT mass spectrometer (ThermoElectron Corporation, San Jose, CA). The LTQ-FT is a hybrid mass spectrometer with a linear ion trap used typically for MS/MS fragmentation (i.e. peptide sequencing) and a Fourier transform ion-cyclotron resonance (FT-ICR) mass spectrometer used primarily for primary MS accurate-mass measurement of peptide molecular ions. The LTQ-FT is equipped with a nanospray ion source (ThermoElectron Corp.).

Approximately 5 to 20 fmoles of tryptic digest or phosphopeptide-enriched samples were dissolved in 5% acetonitrile with 0.1% formic acid and injected onto a C18 nanobore LC column for nano-LC/MS/MS and identification of peptides. The nanobore column was homemade (C18 (Atlantis, Waters Corp); 3 um particle; column: 75um ID x 100 mm length) Atlantis dC18, 3 μm × 75 μm × 100 mm (Waters Corp.). A linear gradient LC profile was used to separate and elute peptides with a constant total flow rate of 350 nL/minute. The gradient consisted of 5 to 70% solvent B in 78 minutes (solvent B: 80% acetonitrile with 0.1% formic acid; solvent A: 5% acetonitrile with 0.1% formic acid).

The LTQ-FT mass spectrometer was operated in the data-dependent acquisition mode controlled by Xcalibur 1.4 software, in which the “top 10” most intense peaks observed in an FT primary scan (i.e. MS survey spectrum) are determined by the computer on-the-fly and each peak is subsequently trapped for MS/MS analysis and peptide fragmentation (sequencing by collision-induced dissociation) in the LTQ linear ion trap portion of the instrument. Spectra in the FT-ICR were acquired from *m*/*z* 350 to 1400 at 50,000 resolution. Mass accuracies are typically within about 2 ppm. The LTQ linear ion trap was operated with the following parameters: precursor activation time 30 ms and activation *Q* at 0.25; collision energy was set at 35%; dynamic exclusion width was set at low mass of 0.1 Da and high mass at 2.1 Da with one repeat count and duration of 10 s.

# Mascot Database Searches

LTQ FT MS raw data files were processed to peak lists with BioworksBrowser 3.2 software (ThermoElectron Corp., San Jose, CA). Processing parameters used to generate peak lists were as follows: precursor mass 351-5500 Da; grouping was enabled allowing 5 intermediate MS/MS scans; precursor mass tolerance 5 ppm, minimum ion count in MS/MS was set to 15, and minimum group count was set to 1.

Resulting DTA files from each data acquisition file were merged, and the Merge data file was searched to identify peptides / proteins against the latest version of NCBInr (*Drosophila* taxonomy sub-database) using MASCOT search engine (Matrix Science Ltd.; version 2.2.1; in-house licensed). Searches were done with tryptic specificity, allowing two missed cleavages, or “non-specific cleavage” and a mass error tolerance of 5 ppm in MS spectra (i.e. FT-ICR data) and 0.5 Da for MS/MS ions (i.e. LTQ linear ion trap).

Variable modifications included in the searches were oxidation of: methionine, histidine, and tryptophan. Identified peptides were generally accepted only when the MASCOT ion score value exceeded 20.

The false discovery rate (FDR) searches were not performed yet with this data.

However, FDR searches are typically calculated using a random-protein sequence “decoy” database created during the search by the MASCOT software. The FDR is calculated as the number of false positives (FP), determined by searching the decoy database, divided by the number of matches (M) in the target “*Drosophila*” database (FDR=FP/M). A significance threshold of p< 0.05 is typically used for FDR calculations. The decoy database consists of random sequences, of the same length of each identified peptide, and average amino acid distribution of the “*Drosophila*” database. FDR from the “decoy” database searches can range, for example, from 0.00% to 5% for different acquisitions or levels of confidence in identified peptides.
